# Drug repurposing for cancer therapy

**DOI:** 10.1038/s41392-024-01808-1

**Published:** 2024-04-19

**Authors:** Ying Xia, Ming Sun, Hai Huang, Wei-Lin Jin

**Affiliations:** 1 Center for Clinical Laboratories, The Affiliated Hospital of Guizhou Medical University, Guiyang, 550004 PR China; 2 The First Affiliated Hospital of Guizhou University of Traditional Chinese Medicine, Guiyang, 550001 PR China; 3 School of Clinical Laboratory Science, Guizhou Medical University, Guiyang, 550004 PR China; 4 Division of Gastroenterology and Hepatology, Department of Medicine and, Department of Oncology, Sidney Kimmel Comprehensive Cancer Center, Johns Hopkins University School of Medicine, Baltimore, MD 21287 USA; 5 Institute of Cancer Neuroscience, Medical Frontier Innovation Research Center, The First Hospital of Lanzhou University, The First Clinical Medical College of Lanzhou University, Lanzhou, 730000 PR China

**Keywords:** Drug development, Cancer therapy

## Abstract

Cancer, a complex and multifactorial disease, presents a significant challenge to global health. Despite significant advances in surgical, radiotherapeutic and immunological approaches, which have improved cancer treatment outcomes, drug therapy continues to serve as a key therapeutic strategy. However, the clinical efficacy of drug therapy is often constrained by drug resistance and severe toxic side effects, and thus there remains a critical need to develop novel cancer therapeutics. One promising strategy that has received widespread attention in recent years is drug repurposing: the identification of new applications for existing, clinically approved drugs. Drug repurposing possesses several inherent advantages in the context of cancer treatment since repurposed drugs are typically cost-effective, proven to be safe, and can significantly expedite the drug development process due to their already established safety profiles. In light of this, the present review offers a comprehensive overview of the various methods employed in drug repurposing, specifically focusing on the repurposing of drugs to treat cancer. We describe the antitumor properties of candidate drugs, and discuss in detail how they target both the hallmarks of cancer in tumor cells and the surrounding tumor microenvironment. In addition, we examine the innovative strategy of integrating drug repurposing with nanotechnology to enhance topical drug delivery. We also emphasize the critical role that repurposed drugs can play when used as part of a combination therapy regimen. To conclude, we outline the challenges associated with repurposing drugs and consider the future prospects of these repurposed drugs transitioning into clinical application.

## Introduction

Cancer remains a leading cause of death worldwide, posing a significant burden on global health.^1,2^ The high incidence of cancer may be caused by several factors, such as genetic mutations, environmental factors, insufficient physical activity, diverse lifestyles, unstable behaviors related to diet, smoking, and alcohol consumption.^3–7^ The current treatment methods for different stages of various cancers include chemotherapy, radiation therapy, and surgical procedures for solid tumors, or a combination of the above.^8^ Although these different treatment modalities can effectively reduce cancer, patients may also experience side effects. Radiation therapy runs the risk of causing DNA damage in surrounding healthy cells, which could potentially lead to new incidences of cancer.^9^ Similarly, although surgical intervention—the primary treatment for solid tumors—significantly improves patient survival, its success rate depends on the expertise of the surgeon and the availability of screening methods, including hospital imaging equipment.^10^ The introduction of chemotherapy was a milestone in cancer treatment. However, prolonged use of chemotherapy drugs, especially those affecting tumor cell metabolic pathways and signal transduction, can influence tumor occurrence, metastasis, drug response, recurrence, drug resistance, and cancer stem cells (CSCs).^11,12^ Therefore, there remains an urgent need to develop novel treatment strategies with high anti-tumor efficacy and minimal side effects.

Traditionally, drug development involves preclinical research and clinical trials. Preclinical studies involve testing the efficacy, toxicity, pharmacokinetics, and pharmacodynamics of drugs in human tumor cells and animal models. Once the therapeutic efficacy of a drug has been determined, the drug moves into the clinical trial phase, which includes Phase I, II, and III human clinical trials, to determine the safety and effectiveness of the drug. As such, it takes 10–15 years and costs $1–2 billion to produce a new drug approved for clinical use. Despite these investments, less than 1% of compounds are expected to enter clinical trials, let alone reach the market.^13–15^ The strategies of drug repurposing involve exploring new therapeutic applications for drugs that have already been approved. Drugs that were originally approved for one indication and have since been studied and used to treat different medical conditions are gaining prominence. This approach is exemplified in the comprehensive review by Kirtonia et al., which underscores the innovative methodologies and potential transformative impact of drug repurposing specifically in the field of oncology.^16^ Drug repurposing has several inherent advantages including a faster and more cost-efficient drug development time due to prior knowledge about the safety, dosage, and toxicity profiles of existing medications. In recent years, the interest in drug repurposing has risen. Successful candidates including chlorambucil and bufulfone were originally developed as alkylating agents based on the toxic chemical warfare agent mustard gas but were later found to be effective for treating leukemias.^17^ Similarly, thalidomide, despite its infamous history of causing severe birth defects, has been repurposed to treat conditions such as leprosy and multiple myeloma.^18^ In addition, arsenic trioxide (a poison) and all-trans retinoic acid (a metabolite of vitamin A) are examples of other chemical compounds^19^ that were approved by the FDA in 2000 for the treatment of acute promyelocytic leukemia. Thus, drug repurposing may be a compelling and viable strategy for enhancing cancer treatment options.^20^ In this review, we embark on a comprehensive examination of drug repurposing as a potential strategy for the treatment of cancer. We begin with an introduction to the definition and background of drug repurposing, setting the stage for its potential use in cancer treatment. To provide a context for the application of repurposed drugs, we offer an in-depth discussion of the epidemiology and current treatment landscape of cancer, including a detailed overview of the 14 updated hallmarks of cancer, which serve as critical targets for novel therapeutic strategies. We then discuss the molecular mechanisms through which repurposed drugs exert their antitumor effects, focusing on their role in regulating different aspects of the tumor microenvironment (TME). Next, we summarize the innovative application of nanomaterials to enhance the delivery of repurposed drugs, shedding light on this expanding area of research. We also identify and discuss the many obstacles and challenges faced during the bench-to-bedside translation of repurposed drugs. Finally, we emphasize the transformative potential of drug repurposing in advancing the field of tumor treatment and highlight its ability to introduce more effective and less toxic therapeutic options for cancer patients.

## Drug repurposing strategies

In summary, drug repurposing can be divided into three stages: identifying the core targets of the disease (hypothesis generation), determining the efficacy of the drug through in vitro and in vivo models, and proceeding to phase II clinical trials in cases where phase I trials have yielded adequate data.^21–23^ The inception stage is critical since hypothesis generation is the key to any drug repurposing endeavor.^24^ Historically, drug repurposing in oncology has largely been driven by either an understanding of the disease pathways or through serendipitous findings. Thus, designing innovative strategies to match existing drugs with newfound applications could increase the success of drug repurposing. Identification of a potential repurposed drug can be made using computational and experimental methods. The experimental approach considers tools such as induced pluripotent stem cell models and function-first phenotypic screenings (or reverse chemical biology),^25,26^ while computational methods use target-centric, knowledge-driven, signature-aligned, pathway-focused, and mechanism-specific strategies.^27,28^ More often, these techniques are synergistically utilized. Notably, high-throughput screening using sophisticated models can identify compounds that mitigate disease symptoms without necessitating pre-existing knowledge about the drug-target interactions.^29,30^ Current computational methodologies, such as merging drug effects with clinical disease signatures and model systems that predict disease-modifying effects, are available for the selection of drug candidates suitable for drug repurposing in cancer. These tools can identify ligands, decode drug ingredient binding schemas, and highlight promising candidates from an expansive list of potential compounds.^27,31,32^ In summary, although the idea of drug repurposing is long-established, it is only recently that technological advances, such as the ones outlined in this article, have led to the development of cutting-edge strategies that can be consciously paired with novel indications.

### Experimental approaches

#### Organoid models of cancer

Organoids are classified as “stem cell-containing self-organizing structures”, while tumoroids are a special type of cancer organoid.^33^ Organoids are in vitro tissues that originate from human stem cells, organ-specific progenitor cells, or even disassociated tumor tissues and are cultured in specialized ECM-based medium with relatively high success rates. Tumoroids mimic the primary tissue in both architecture and function and retain the histopathological features, genetic profile, mutational landscape, and even responses to therapy.^34^ The use of tumoroids is expanding, and their utility for basic research and early steps of drug development has been recognized.^35^ Cisplatin, for example, has been found to be less effective in patient-derived organoids (PDOs) generated from non-small cell lung cancer (NSCLC) tissues than from cell lines, highlighting the ability of patient-derived material to provide important information on potential resistance mechanisms.^36^ With respect to gastrointestinal cancers, several investigations have harnessed PDOs as tools to evaluate drugs and identify potential therapeutic routes.^37,38^ Such models have adeptly mirrored the feasibility of tumoroids in the accurate recapitulation of KRAS-mutant metastatic rectal cancer with microsatellite stability after hepatic resection and treatment with neoadjuvant combination chemotherapies in colorectal cancer (CRC),^39^ as well as gauged drug reactions in hepatocellular carcinoma (HCC)^40,41^ and mimicked treatment resistance patterns observed in esophageal squamous cell carcinoma.^42^

**Fig. 1:**
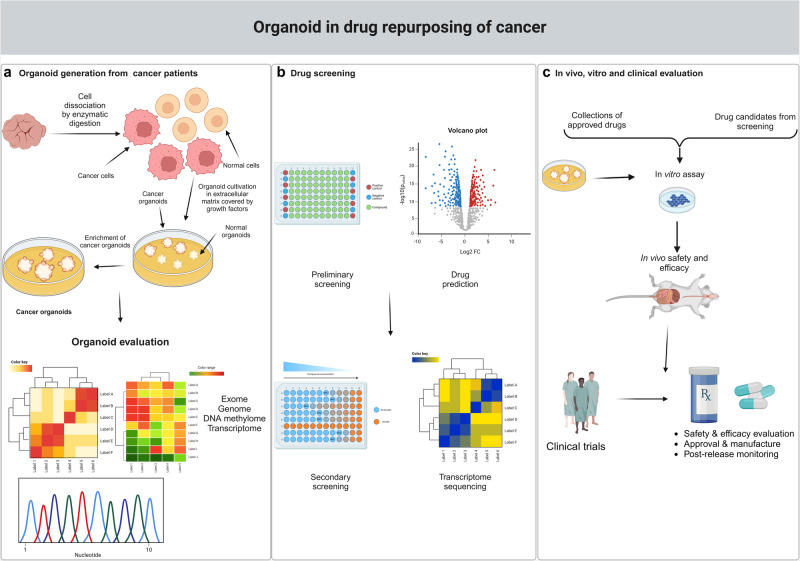
Tumoroids model in drug repurposing. **a** Schematic showing the generation of patient-derived organoids (PDOs) from a cancer biopsy: enzymatic digestion, embedding in extracellular matrix, addition of growth medium and cancer tumoroids enrichment by media compound withdrawal and/or addition of mutation related inhibitors. **b**, **c** The tumoroid model is used to screen drug repurposing candidates, resulting in the identification of drugs for preclinical and clinical testing. This figure was created with Biorender.com

In addition, tumoroid models present a distinct advantage in cancer drug screening due to their ability to emulate the structure, gene expression patterns, and essential characteristics and functionalities of their originating organs (Fig. 1). For example, SMAC mimetics such as LCL161 have been studied in hepatic metastatic rectal cancer,^39^ while novel CDK7 inhibitors like YPN-005 have been analyzed in SCLC tumoroid systems.^43^ A high-throughput screening, based on the interaction between patient-derived breast cancer organoids and tumor-specific cytotoxic T cells, identified three epigenetic inhibitors - BML-210, GSK-LSD1, and CUDC-101—that displayed significant antitumor effects.^44^ In addition, the drug atorvastatin was found to inhibit angiogenesis in a dose-dependent manner through the downregulation of vascular endothelial growth factor (VEGF), CD31, and Bcl-2 in a co-culture of glioblastoma organoids and human umbilical vein endothelial cells in fibrin gels, indicating that atorvastatin may be a promising drug for the treatment of glioblastoma.^45^ Finally, a 2019 research study demonstrated the predictive potential of PDOs for personalized medicine, using a biobank of PDOs sourced from cancer patients participating in phase I/II clinical trials.^46^

Tumoroids accurately model human primary tumors, positioning them as an invaluable platform for both foundational research and translational medicine. This includes their use in cancer models to study tumorigenesis and cancer progression, as well as in the prediction of drug responses, treatment optimization, and the discovery of novel anticancer therapeutics. Despite these advantages, current tumoroid systems are not without limitations. A primary concern is that the tissue samples used to create organoids represent only a fraction of the entire tumor. Given the substantial heterogeneity inherent in tumors, the reliability of using small tissue samples to effectively represent the entire tumor mass is questionable. Thus, caution is required when extrapolating tumoroid data to the whole tumor. In addition, tumoroid models often lack key non-tumorous cellular components, such as mesenchymal tissues, neural cells, and immune cells. The absence of these cell types in tumoroids limits their ability to fully mimic the complex structure and functionality of their corresponding organs. In particular, the imprecise modeling of the tumor immune environment significantly impedes the utility of tumoroids in both translational and precision medicine applications. Another major challenge is the vascularization of tumoroids.^47^ Effective vascularization is crucial for accurately replicating tumor biology, yet it remains an unresolved issue in the development of tumoroids. Furthermore, standardization of specific tumoroid culture conditions is essential for enhancing reproducibility on a large scale and facilitating the application of tumoroid technology in high-throughput drug screening. The specific culture conditions required for cancer organoids, if not meticulously managed, can lead to a reduction in the intrinsic diversity within tumors over prolonged cultivation periods. In summary, while tumoroid models offer significant insights and advances in cancer research and treatment development, addressing these challenges is crucial for maximizing their potential and applicability in advanced cancer research and personalized medicine.^48,49^

#### Phenotypic analysis

Several drugs that exhibit potent off-target effects (side effects) in cancer are also worth exploring. These off-target effects can be viewed as new indications of the drug for other diseases (phenotypic analysis).^25^ Phenotypic screening is a strategy that analyzes biology-associated (phenotypic) effects in given models such as animals, cells, or organisms to help identify the targets of candidate drugs.^25,50^ A 96-well or 384-well format is typically utilized for in vitro phenotypic screening.^51^ Previous studies have identified repurposed drugs by conducting high-throughput cell-based screening, using some ‘classical’ hallmarks of in vitro phenotypes including sustained proliferation, increased angiogenesis, and resistance to cell death.^25^ For example, Jacquemet et al. utilized a phenotypic screen to identify FDA-approved calcium channel blockers as potent inhibitors of filopodia formation in cancer cells. Cancer cells expressing MYO10-GEP were treated with different drugs from the compound library. From this screen, L-type calcium channel blockers, such as amlodipine besylate, felodipine, diclomanidipine, and cilidipine, were found to inhibit filopodia formation and prevent cancer cell invasion, thereby highlighting the importance of L-type calcium channels in regulating calcium entry and filamentous pore stabilization.^52^ Thus, phenotypic screening and identifying drug candidates with yet-to-be-identified targets can economize both time and resources in the drug discovery process, as well as minimize premature clinical trial setbacks.

### Computational approaches

Computational methodology has emerged as a powerful tool in the field of drug repurposing.^53,54^ Our understanding of the mechanisms and modes of action within oncology has deepened substantially with the increase in omics technologies coupled with breakthroughs in big data analytics, machine learning, and computational algorithms. These computational techniques grant expansive access to both disease-centric and drug-centric data.^55,56^ Several computer-assisted drug repurposing strategies such as molecular docking, network analysis, data mining, similarity analysis, machine learning, and transcriptional signature techniques, are readily available to researchers.^57,58^ Through these computational approaches, we can delve further into the anticancer prospects of drug repurposing and provide disease-related data for the repurposing of drugs.^59,60^ The identification of oncogenic pathway inhibitor activity via computer-aided drug repurposing approaches also represents a robust method.^61,62^ Researchers can exploit multiple databases for extensive analysis of drug bioinformatics (Table 1). And repurposed drugs identified by network-centric systems biological approaches are shown in Table 2. These repositories not only amplify the therapeutic potential of repurposed drugs across various diseases,^63,64^ but also strengthen chemotherapeutic strategies, providing novel strategies to reduce the development of resistance and tailor treatments to maximize patient-specific outcomes.^65,66^

**Table 1:** The investigated databases

Category	Name	Description	Links	Ref.
Genome/Target	UCSC	A database with rapid and reliable display of any requested portion of the genome at any scale, together with several dozen aligned annotation tracks	https://genome.ucsc.edu/	^400^
	GenBank	A database that contains publicly available nucleotide sequences for 400 000 formally described species	https://www.ncbi.nlm.nih.gov/genbank/	^401^
	Connectivity Map	A database with genome-wide transcriptional expression data	https://portals.broadinstitute.org/cmap/	^402^
	Ensembl	A database provides high-quality genome annotation across chordate species through a comprehensive set of methods	https://www.ensembl.org/index.html	^403^
	Gene Ontology	A database of functional genomics	http://geneontology.org/	^404^
Proteomics/Pathway	UniProt	A database with important collection of protein sequences and their annotation	https://www.uniprot.org/	^405^
	UniGene	A database with annotations for a majority of the human transcripts	https://www.ncbi.nlm.nih.gov/unigene/	^406^
	UniRef	A database with cluster of protein sequence space based on sequence similarity	https://www.uniprot.org/uniref/query/	^407^
	KEGG	A database with genome sequencing and high-throughput functional genomics experiments molecular datasets	https://www.kegg.jp/	^408^
	STRING	A database with critical assessment and integration of protein-protein interactions	https://cn.string-db.org/	^409^
	BiGRID	A database with archive of genetic and protein interactions	https://thebiogrid.org/	^410^
Proteomics/Pathway	HAPPI	A comprehensive database covering human PPI data	http://discovery.informatics.uab.edu/HAPPI	^411^
	Reactome	A database with critical assessment and integration of protein-protein interactions	https://reactome.org/	^412^
	The Human Protein Atlas	A database with critical assessment and integration of protein-protein interactions	http://www.proteinatlas.org/	^413^
Disease Database	The Cancer Genome Atlas	A database with genomic data for more than 30 cancer types	https://www.cancer.gov/about-nci/organization/ccg/research/structural-genomics/tcga	^414^
	Cancer Cell Line Encyclopedia	A database pf large, annotated cell-line collections	https://portals.broadinstitute.org/ccle	^415^
	OMIM	A database with comprehensive compendium of information on human genes and genetic disorders	https://www.omim.org/	^416^
	GEO	A database of gene expression profiles	https://www.ncbi.nlm.nih.gov/geo/	^417^
	LINCS	A database of gene expression data and how human cells respond to different genetic and environmental conditions	https://lincsproject.org/	^418^
Phenome	RepoDB	A repository of approved and failed drug-disease associations	http://apps.chiragjpgroup.org/repoDB/	^419^
	Clinical Trials	A database of publicly and privately funded clinical studies	https://www.clinicaltrials.gov/	^420^
	Drugs @FDA Database	A database of FDA approved drugs and related information	https://www.accessdata.fda.gov/scripts/cder/daf/index.cfm	^421^
	DrugBank	A database of drug-related information	https://go.drugbank.com/	^421^
Drug database	STITCH	A database integrates data sources for 430 000 chemicals into a single, easy-to-use resource	http://stitch.embl.de/	^422^
	SFINX	A database with drug-drug interactions and related information	http://sfinx.ugent.be/	^423^
	TTD	A database of drug-related information such as 3D structure, therapeutic class, and clinical development status	http://db.idrblab.net/ttd/	^424^
	SIDER	A database of adverse drug reactions related to drugs.	http://sideeffects.embl.de	^425^
	Drug versus Disease	A database with comparison of drug and disease gene expression profiles from public microarray repositories	https://omictools.com/dvd-tool	^426^

**Table 2:** Drug repurposing based on the studies using network-based approaches

Drug	Methods	Disease	Reference
Metformin	DSPathNet	Breast, pancreas and prostate cancers	^427^
	Mapping the proteomic profile onto SIGNOR database	Breast cancer	^428^
	A structure-based method to identify proteome-wide molecular targets of metformin	Various cancer types	^429^
	SMiR-NBI	Breast cancer	^430^
	SDTNBI	Various cancer types	^431^
Statins	Weighted gene co-expression network analysis and Cmap querying	Gastric cancer	^432^
	Cell cycle profiling	Various cancer types	^433^
	viPEr and PEANuT	Various cancer types	^434^
	MDP	Various cancer types	^435^
Proton pump inhibitor	Molecular docking	Various cancer types	^436^
	Molecular docking	Pancreatic cancer	^437^
Disulfiram	b-SDTNBI	Breast cancer	^438^

## Drug repurposing: candidates for the therapeutic targeting of hallmarks of cancer

**Fig. 2:**
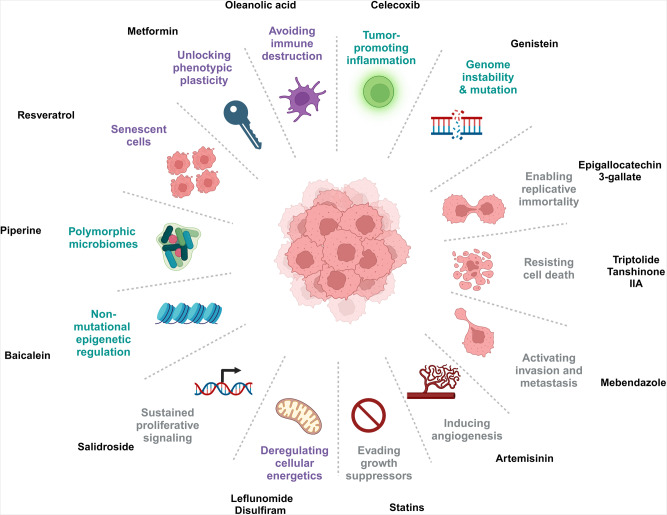
Diverse cancer hallmarks targeted by repurposed non-oncology drugs. Repurposed non-oncology candidates have shown great promise against cancer by targeting different hallmarks of cancer including sustaining proliferative signaling, evading growth suppressors, resisting cell death, enabling replicative immortality, inducing angiogenesis, activating invasion and metastasis, genome instability and mutation, tumor-promoting inflammation, reprogramming energy metabolism, evading immune destruction, unlocking phenotypic plasticity, non-mutational epigenetic regulation, polymorphic microbiomes, and senescent cells. This figure was created with Biorender.com

The hallmarks of cancer are fundamental characteristics that drive the development and progression of cancer. Initially proposed by Hanahan and Weinberg in 2000, this concept has since been expanded to encompass 14 distinct hallmarks of cancer.^67,68^ Understanding these hallmarks is pivotal for developing effective strategies for cancer prevention, diagnosis, and treatment. Indeed, targeting the hallmarks of cancer has emerged as a promising approach towards the development of novel therapies that strike at the root causes of the disease, offering the potential for durable and transformative patient benefits.^69^ This approach uses the known safety and pharmacologic profiles of existing drugs to potentially accelerate the development of effective and affordable cancer therapeutics. Here, we systematically review the current progress in this area of research and provide representative examples for each of the hallmarks of cancer (Fig. 2), specifically describing how drug repurposing could be used to target these hallmarks. This provides a comprehensive background for further investigations into the potential of drug repurposing in cancer treatment, and a strong theoretical foundation that could guide the identification of promising new drug candidates.

### Inhibiting proliferative signaling

Cancer cells are characterized by their inherent ability to sustain chronic proliferation, which effectively enables them to become self-sufficient in growth signaling and to control their own fate. Uncontrolled proliferation is primarily facilitated by deregulation of the production and release of growth-promoting signals.^69^ Growth-promoting signals are predominantly transmitted via growth factors that bind to cell-surface receptors, which typically contain intracellular tyrosine kinase domains.^70^ The phosphatidylinositol 3-kinase/protein kinase B (PI3K/AKT), mammalian target of rapamycin (mTOR),^71^ and mitogen-activated protein kinases/extracellular signal-regulated kinase (MAPK/ERK) pathways have all been implicated in sustaining proliferative signaling. However, while these pathways are significant, they represent only a subset of the pathways involved in cancer cell proliferation.^72,73^ In the current landscape of cancer treatment, an increasing number of drugs, originally developed for non-oncological conditions, are being repurposed to target these signaling pathways. Such approaches exemplify the creative and adaptive strategies being undertaken to combat the complexity and adaptability of cancer.

#### Salidroside

Salidroside, an active compound isolated from the dried roots, rhizomes, and entire plants of *Rhodiola rosea*, has attracted recent attention due to its wide-ranging pharmacological activities, including anti-hypoxic, anti-aging, immune-enhancing, and anti-fibrotic properties.^74–78^ Among these diverse effects, the ability of salidroside to act as an anticancer agent is of interest. For example, salidroside treatment has been shown to inhibit the proliferation of nasopharyngeal carcinoma (NPC) cells, including the CNE2 and HONE cell lines, through regulation of the miR-4262/GRP78 axis.^79^ Similarly, Liu et al. showed that salidroside suppressed proliferation, colony formation, and migration of the PC3 and DU145 prostate cancer cell lines in a dose-dependent manner by inhibiting the PI3K/AKT pathway.^80^ In addition, salidroside was found to impede cancer cell proliferation in HeLa cells and a subcutaneous HeLa-ADR-luc (doxorubicin-resistant derived HeLa-cell lines) cell xenograft mouse model through the activation of apoptosis and inhibition of the PI3K/ Akt/HIF-1α signaling pathway.^81^ These preclinical findings suggest that repurposing salidroside as a potential anticancer agent is worthwhile. However, due to the limited clinical research on salidroside, there remains a critical need for further clinical trials to validate these effects and facilitate the translation of salidroside into a viable treatment option in clinical practice.

### Inducing cell death

**Fig. 3:**
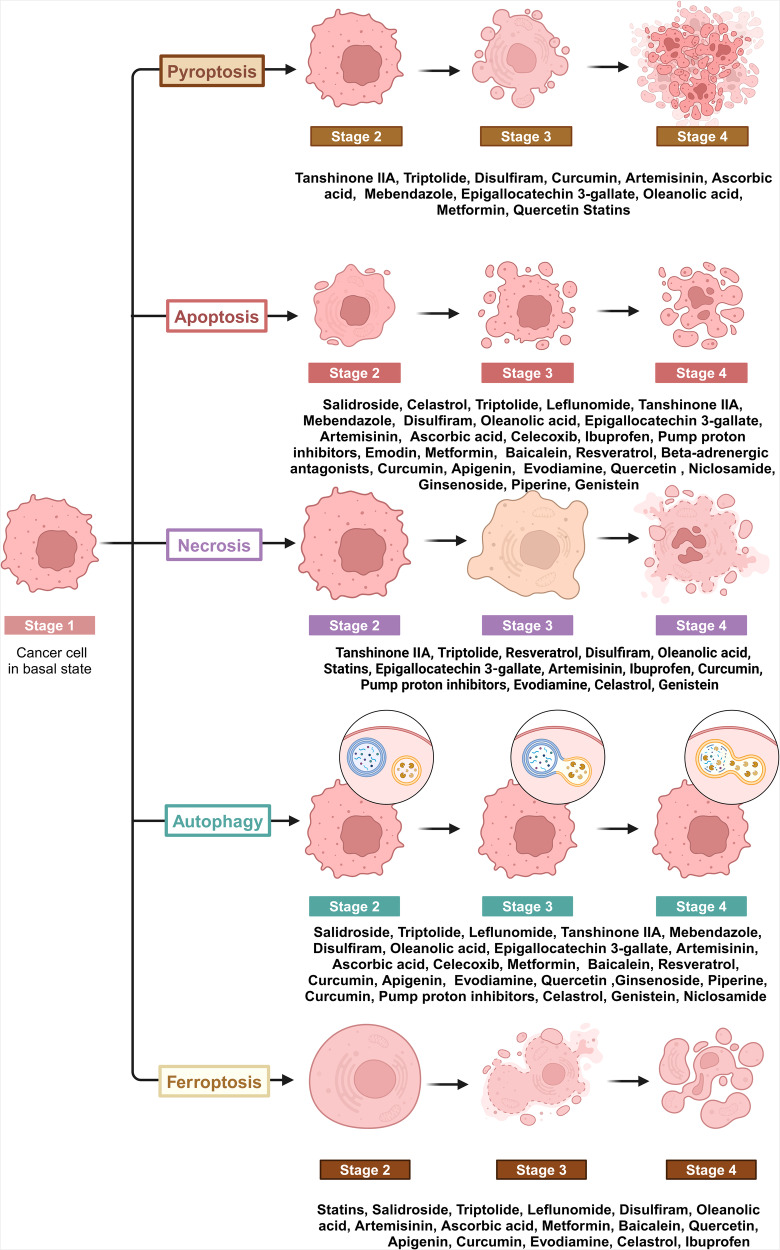
Inducing cell death in cancers by repurposed non-oncology drugs. Regulated cell death (RCD) is a critical and active process that is controlled by specific signal transduction pathways and can be regulated by drug interventions. Repurposed non-oncology candidates can exert anticancer effects by inducing classical apoptosis and other RCD processes, such as ferroptosis, autophagy, necroptosis and pyroptosis. This figure was created with Biorender.com

Cell death is a critical process in biological systems that is not only essential for the maintenance of correct physiological development and tissue homeostasis, but also acts as a natural defense mechanism against tumor formation. Currently, cell death can be classified as accidental cell death (ACD) and regulated cell death (RCD). Unlike ACD, which is generally an uncontrolled process, RCD is more organized and involves genetically encoded molecular mechanisms that help to maintain a stable internal environment.^82^ RCD can be further subdivided into apoptotic and non-apoptotic subcategories.^83^ Since RCD has a fundamental role in cellular regulation and can act as a barrier to tumorigenesis, targeting RCD pathways through drug repurposing may be a promising strategy to impede the development and progression of tumors (Fig. 3). Accumulating evidence has highlighted the potential of using repurposed drugs to restrict tumor growth. These drugs have been shown to activate various RCD pathways, including apoptosis, necroptosis, pyroptosis, autophagy, ferroptosis, and cuproptosis. It is important to note, however, that since cuproptosis is a newer area of study, no associated preclinical trials are currently underway.

#### Triptolide

Triptolide (TPL) was first isolated in 1972 from a perennial vine-like herb called Thunder God Vine.^84^ Since then, its mechanism of action and pharmacological properties have been extensively researched to reveal prominent anti-rheumatic, anti-bacterial, anti-inflammatory, and immunomodulatory activities. More recent studies have also demonstrated that TPL possesses anti-tumor properties.^85–88^ Specifically, cells exposed to TPL were found to undergo non-apoptotic cell death. Treatment with TPL resulted in morphological alterations characterized by cytoplasmic swelling and membrane disruption, as well as a marked elevation in the mRNA and protein expression of gasdermin E (GSDME) and GSDMB. Selective inhibition of GSDME was found to counteract TPL-induced cell death and mitigated cytoplasmic swelling and membrane disruption.^89^ In addition to pyroptosis, Wang and colleagues also found that the use of TPL-loaded polyethylene glycol (PEG) nanocarriers induced necrosis and enhanced the sensitivity of MIA PaCa-2 (pancreatic ductal adenocarcinoma) tumors to gemcitabine.^90^ Furthermore, TPL has been shown to induce autophagy-mediated caspase-independent cell death in tumor cells. One study showed that in prostate cancer cells, TPL-based drugs stimulated the release of free calcium, promoted endoplasmic reticulum stress and induced activation of the CAMKKb-AMPK signaling pathway, leading to inhibition of mTOR and activation of beclin-1 and Unc-51-like kinase 1 (ULK1), thereby promoting autophagy.^91^ Currently, a clinical trial has investigated the safety and anticancer efficacy of the TPL derivative F60008 in patients with advanced solid tumors. However, due to significant variability among patients and high toxicity in some, F60008 cannot be considered as an appropriate derivative of TPL for cancer patients.^92^ Minnelide, another derivative of TPL, is presently under investigation for the treatment of refractory advanced pancreatic adenocarcinoma, either alone or in combination with paclitaxel (PAX) (NCT04896073 and NCT03117920).

#### Tanshinone IIA

Tanshinone IIA, an active compound extracted from *Salvia Miltiorrhiza* (Danshen), is traditionally used as a low-cost and safe treatment for various ailments, including cardiovascular and cerebrovascular diseases.^93^ However, more recently, Tanshinone IIA has been shown to induce various types of cell death in cancer cells and may therefore be a potential anti-tumor agent. For example, Tanshinone IIA has been shown to exert anti-neoplastic effects in renal cell carcinoma (RCC) cells through the down-regulation of β-catenin, which results in the formation of autophagic vacuoles such as autophagosomes and autolysosomes, increased apoptosis, and the induction of autophagic cell death.^94^ In addition, Tanshinone IIA has been shown to induce ferroptosis in gastric cancer (GC) cells through p53-mediated down-regulation of solute carrier family 7 member 11 (SLC7A11), leading to elevated levels of reactive oxygen species (ROS), lipid peroxidation, and iron accumulation, and effectively inhibiting tumor cell growth both in vitro and in vivo.^95^ Interestingly, Lin et al. demonstrated that Tanshinone IIA might also induce necroptosis in human HCC by promoting the formation of a necrosomal complex composed of receptor-interacting protein 1 (RIP1)/RIP3.^96^ Due to its multifaceted effects on cell death pathways, minimal resistance to its targets, and traditional use as a safe compound, Tanshinone IIA is emerging as a compelling candidate for anti-tumor therapies. Thus, a deeper understanding of its mechanisms of action in the context of cancer treatment is critical.

### Regulation of cellular metabolism

The rapid proliferation of cancer cells is sustained through corresponding adaptations in tumor metabolism, which involve the activation or modification of metabolic pathways to harness more energy.^97^ Deregulating cellular energetics has recently been added as a new hallmark of cancer.^98^ Current research on metabolic reprogramming has primarily focused on the aberrant activation of the PI3K/AKT/mTOR pathway, as well as activation of oncoproteins such as MYC, RAS, pyruvate kinase M2 (PKM2), and hypoxia-inducible factor 1 (HIF-1). In addition, the role of mutations or deactivation of tumor suppressor genes, including P53 and phosphatase and tensin homolog (PTEN) are being explored.^97,99–101^ In the past decade, only a few metabolism-based cancer drugs have been successfully developed, some of which are in or nearing clinical trials. Meanwhile, repurposed drugs have been extensively examined in preclinical studies as a potential means of targeting key pathways in malignant metabolism. Thus, using repurposed drugs to target essential pathways in tumor metabolism presents a promising therapeutic strategy.

#### Leflunomide

Leflunomide, an immunomodulatory drug primarily prescribed for rheumatoid arthritis and psoriatic arthritis, has seen a resurgence in research interest due to its potential anticancer properties.^102^ Mechanistically, the metabolic effects of leflunomide stem from its active metabolite, A77 1726, which inhibits the mitochondrial enzyme dihydroorotate dehydrogenase (DHODH). In the 1990s, leflunomide was evaluated as an epidermal growth factor receptor (EGFR) inhibitor with potential anticancer applications.^103,104^ Recent studies have highlighted its potential in treating breast and prostate cancer.^105,106^ In addition, Yamaguchi and colleagues proposed that leflunomide-mediated pyrimidine synthesis could be a therapeutic target for mitigating the metastatic progression of CRC. Further studies have suggested that the action of leflunomide on DHODH, combined with its disruption of de novo pyrimidine biosynthesis, can induce apoptosis in CRC cells that express transcriptionally active P53. These effects appear to be linked to inhibition of the electron transport chain complex III.^107^ Leflunomide has also been shown to suppress melanoma growth by impeding the effective transcription elongation of requisite genes. In human A375 melanoma cells, for example, nucleotide depletion by leflunomide reduced the chromatin occupancy of the RNA helicase protein DDX21. Combination therapy using leflunomide with checkpoint kinase 1 (CHK1) inhibitors has shown enhanced efficacy in reducing the growth of P53-deficient breast tumors and inducing cell apoptosis compared to treatment with leflunomide alone.^108^ Furthermore, in a phase I clinical trial aimed at refractory multiple myeloma, leflunomide displayed manageable side effects, with disease stabilization occurring in 9 of the 11 patients (NCT: NCT02509052). Collectively, these findings indicate that leflunomide is a promising candidate for broader cancer therapy applications.

#### Disulfiram

Disulfiram (DSF), also known by its trade name Antabuse, was originally approved by the FDA in 1951 as a treatment for alcoholism.^109^ The anticancer properties of DSF were serendipitously discovered in 1977 when Lewison reported the drug’s potential to inhibit bone metastasis in breast cancer patients.^110^ This initial observation has since been substantiated through extensive research, including analyses of Danish demographic and health registries that observed lower mortality rates for colon, prostate, and breast cancers among ongoing DSF users compared to former users.^111^ Notably, DSF has recently attracted attention for its potent anticancer effects and its capacity to modulate cellular energy metabolism. For example, Du et al. found that DSF inhibited the glycolysis of cancer cells in a copper-dependent manner. Furthermore, a combination of DSF and copper was shown to significantly reduce the expression levels of key molecules, including S6K1, MYC, and their downstream targets, glucose transporter 1 (GLUT1), PKM2, and lactate dehydrogenase A (LDHA), which are integral to the regulation of critical cellular processes, such as apoptosis, cell differentiation, and metabolism.^112^ DSF has also been reported to augment oxidative metabolism within thyroid cancer cells, primarily by increasing ROS production, which, in turn, triggers apoptosis in an ROS-dependent manner.^113^ Given its anti-tumor properties and metabolic modulating potential, DSF is a promising drug to include in combination therapy strategies using repurposed drugs.

### Activating antitumor immunity

In the early stages of tumorigenesis, the body’s lymphocytes, including cytotoxic T cells and natural killer (NK) cells, actively target and aim to eliminate emerging cancer cells through the secretion of perforin and granzyme to induce apoptosis or through activation of the death ligand/death receptor pathway. Perforin creates pores in the membranes of the target cells, allowing granzymes to enter and subsequently initiate apoptosis.^114^ However, during tumor progression, the immunosuppressive mechanisms within the TME become more pronounced. For example, tumor cells may begin to express programmed death ligand 1 (PD-L1), a ligand that binds to the PD-1 protein on NK cells and T cells, thereby inhibiting their activity. In addition, the emergence of suppressive immune cellular populations further limits the effectiveness of the body’s natural antitumor immunity.^115–117^ In light of these challenges, an increasing number of immunotherapies have been developed and used to treat tumors. Notable among these are adoptive cell transfer (ACT) and immune checkpoint inhibitors (ICIs), both of which aim to strengthen the body’s immune response to cancer. Despite their potential, these strategies are not universally effective across all patient populations. Thus, there is growing interest in identifying repurposed drugs that have the potential to activate antitumor immunity, thereby providing additional strategies to enhance cancer treatment outcomes.

#### Oleanolic acid

Oleanolic acid (OA, 3β-hydroxyolean-12-en-28-oic acid) is a pentacyclic triterpenoid derived from the *Oleaceae* family that is prevalent in many dietary and medicinal plants and possesses anti-diabetic,^118^ anti-bacterial,^119^ anti-parasitic,^120^ and anticancer properties.^121^ The recent interest in OA is largely due to its effects on antitumor immunity, which position it as a potential candidate for cancer treatment through drug repurposing. For example, OA was found to promote the balance of regulatory T cells (Tregs)/Th17 cells in GC by targeting interleukin-6 (IL-6) via the miR-98-5p pathway.^122^ OA has also emerged as an epigenetic modulator in immunotherapy for cancer. OA was shown to inhibit the IL-1/NF-κB/TET3 axis in cancer cells, resulting in DNA hypomethylation and the suppression of PD-L1, thereby strengthening the robust T-cell defense mechanism.^123^ In addition, synthetic derivatives of OA, such as CDDO-Im, have been shown to block the EGFR/signal transducer and activator of transcription 3 (STAT3)/Sox-2 signaling pathway in tumor-associated macrophages (TAMs), which have been implicated in promoting breast cancer proliferation and metastasis.^124^ Thus, OA may be a potential therapeutic agent for the treatment of cancer. In conclusion, although clinical studies examining the role of OA in cancer are limited, existing preclinical evidence indicates that OA may be a suitable drug candidate to treat cancer, and may have the potential to increase the therapeutic outcomes of present-day immunotherapies.

### Reactivating growth suppressors

Tumor suppressor genes, such as P53 and retinoblastoma protein (RB), are crucial regulators in cancer progression.^125–127^ Specifically, P53 acts as a sentinel, responding to intracellular disturbances like metabolic and oxidative stress, and inducing cell cycle arrest until the TME returns to a balanced state.^128,129^ However, some tumors are able to bypass these suppressor genes or deactivate pivotal tumor suppressors. In these cases, repurposed drugs may be able to target cancer cells that have avoided suppressor gene regulation, providing potential therapeutic benefits.

#### Statins

Statins lower circulating blood lipids including low-density lipoprotein (LDL) cholesterol through the competitive inhibition of 3-hydroxy-3-methyl-glutaryl-coenzyme A reductase (HMGCR), an enzyme that facilitates the conversion of HMG-CoA to mevalonic acid, which is a crucial step in cholesterol biosynthesis. By inhibiting this process, statins not only reduce cholesterol production but also impact by-products essential for cancer cell growth, thereby demonstrating their potential as anticancer agents.^130,131^ Of the statins, simvastatin is particularly interesting due to its anticancer applications, which have been observed in various cancer types and are largely mediated through activation of mutant P53. Specifically, simvastatin was found to reduce the migratory and invasive abilities of human epithelial MDA-MB-231 breast cancer cells in vitro by increasing mutant P53 expression and repressing expression of the stem cell marker CD44, which is essential for cell migration. Similarly, in MDA-MB231 mouse xenograft models, simvastatin treatment led to elevated P53 and reduced CD44 expression levels.^132^ In parallel studies, Miyajima and colleagues found that simvastatin and fluvastatin activated the transcriptional function of P53 by suppressing TAZ protein expression. When used in combination with nutlin-3, simvastatin was found to reduce cell viability through the suppression of MDM2 and activation of wild-type P53.^133^ Moreover, certain statins, notably simvastatin and atorvastatin, have been shown to downregulate MCM7 and RB expression. These statins have been linked to an increase in chromosomal abnormalities in RB-deficient tumor cells, hinting at their potential application for the treatment of RB-deficient tumors.^134^ Ongoing clinical trials are currently examining the efficacy of simvastatin against various cancers, including breast (NCT00807950, NCT05550415), gastric (NCT01099085, NCT03086291), colorectal (NCT01238094), and bladder (NCT02360618) cancer. Although the specifics of its mechanism, optimal dosage, and compatibility with other anticancer drugs remain unclear, we anticipate the emergence of novel strategies employing statins in cancer treatment in the future.

### Interfering with replication

Unlike normal cells, cancer cells display elevated levels of the specialized DNA polymerase, telomerase, which provides tumor cells with the unique ability to evade the usual cellular growth and division constraints dictated by senescence and crisis/apoptosis mechanisms.^135,136^ In addition, human telomerase reverse transcriptase (hTERT) plays an integral role in cancer signaling pathways as a transcriptional regulator, orchestrating the activation of crucial genes that are vital for tumor proliferation and survival. The noncanonical roles of hTERT in cancer progression involve the WNT/β‐catenin^137,138^ and nuclear factor‐κB (NF-κB) signaling pathways.^139,140^ hTERT-dependent transcription mediated through these pathways allows tumor cells to have unlimited replication capabilities. Thus, telomerase has emerged as a compelling target for repurposed drugs, potentially bolstering the efficacy of cancer therapies.

#### Epigallocatechin 3-gallate

Epigallocatechin 3-gallate (EGCG), a compound synthesized from epicatechin and gallic acid, has garnered considerable attention in the scientific community due to its multifaceted biological and pharmacological properties. These include its anti-oxidant, anti-inflammatory, anti-angiogenic, anti-proliferative, pro-apoptotic, and anti-metastatic functions. Numerous studies, both in vitro using various cancer cell lines and in vivo in animal models, have consistently demonstrated the ability of EGCG to inhibit the initiation, promotion, and progression of different types of tumors.^141–145^ The ability of EGCG to act as a potent inhibitor of telomerase, an enzyme associated with cellular aging and cancer, is of particular interest. For example, EGCG was shown to induce apoptosis in Hep-2 cells in a dose-dependent manner through the inhibition of telomerase. Similarly, EGCG was found to induce apoptosis in T47D breast cancer cells via inhibition of the telomerase and PI3K/AKT pathways and simultaneous upregulation of the P53 and Bax/Bcl-2 pathways. Impressively, EGCG was able to induce apoptosis in cancer cells without manifesting notable toxicity to healthy cells.^146^ Recently, Dong and colleagues reported the nuanced relationship between EGCG and the WNT/β-catenin signaling pathway by demonstrating that overexpression of β-catenin could either augment or reduce the anticancer effects of EGCG.^147^ In addition, another study found that EGCG was involved in reducing the mRNA expression and transcriptional activity of β-catenin in wild-type P53-expressing KB cells. When used in combination with gemcitabine, EGCG was found to exert stronger inhibitory effects on β-catenin and N-cadherin in pancreatic cancer cells.^145^ Given these promising insights, a phase I clinical trial (NCT00516243) has been initiated that targets women with hormone receptor-negative stages I-III breast cancer and aims to explore the safety and effectiveness of EGCG. Concurrently, several clinical trials for CRC (NCT02321969 and NCT01360320) are also in progress. However, while these studies are promising, the potential therapeutic application of EGCG in cancer treatment is still restricted by its limited bioavailability.

### Decreasing angiogenesis

An “angiogenic switch” is activated temporarily in normal tissue during physiological processes such as wound healing to promote angiogenesis, which accelerates tissue repair. In contrast, during tumor progression, the “angiogenic switch” is activated and remains switched on. Persistent activation promotes the formation of new blood vessels, which not only sustain the growth of neoplastic tissues but also paves the way for tumor invasion and metastasis.^148,149^ The induction of chronic angiogenesis is now acknowledged as a hallmark of cancer. Many of the antiangiogenic agents currently in use, such as bevacizumab, target VEGF or are tyrosine kinase inhibitors that target VEGF receptors (VEGFRs). However, these agents have limitations. For example, sometimes these drugs induce states of stress resistance, which reduce their efficacy. Overall, the strategy of inhibiting angiogenesis via drug repurposing has recently become an area of interest and is worthwhile exploring as a potential target.

#### Artemisinin

Artemisinin, derived from the plant *Artemisia annua*, is a sesquiterpene lactone with a distinct peroxide linkage that has been used as an anti-malarial agent.^150^ Dihydroartemisinin (DHA), a reduced lactol semi-synthetic derivative of artemisinin, boasts an impressive safety record. Interestingly, preclinical and clinical studies have shown that both artemisinin and DHA possess promising anticancer properties in various therapeutic strategies. The endoperoxide bridge, the active moiety of artemisinin derivatives, is cleaved in DHA in the presence of iron ions, leading to the release of cytotoxic ROS, which is thought to be the pivotal mechanism behind the anti-malarial and antitumor properties of DHA.^151^ In addition, neither artemisinin nor DHA have shown substantial toxicity towards normal cells, highlighting their potential to act as suitable anticancer candidates. Recently, an increasing number of studies have demonstrated that DHA also exhibits multi-target anti-angiogenic effects by modulating various angiogenesis signaling pathways. The ability of DHA to markedly inhibit proliferation, migration, and tube formation in human umbilical vein endothelial cells (HUVECs) has been recently reported, while Wang and colleagues found that DHA not only reduced angiogenesis in pancreatic cancer cells but also suppressed NF-κB-DNA binding activity, subsequently downregulating pro-angiogenic genes.^152^ In addition, DHA has been shown to trigger autophagy in HUVECs through suppression of the AKT/mTOR signaling pathway. Interestingly, DHA was also found to enhance VEGFR1 expression through upregulation of ETS-1 transcription factor. With such promising preliminary findings, a Phase II clinical trial (NCT03402464) is currently underway to examine the combined efficacy of DHA with a standard chemotherapy agent, erlotinib, specifically for patients diagnosed with EGFR-mutated lung adenocarcinoma. While these studies have shed new light on the potential of DHA to act as an anticancer agent, more comprehensive clinical trials are necessary in the future to solidify its place in cancer therapy.

### Suppressing invasion and metastasis

Tumor invasion and metastasis contribute to increased tumor-related mortality. Central to these processes is the aberrant activation of the epithelial-mesenchymal transition (EMT) in cancer cells. EMT is regulated by a complex interplay of signaling pathways, including but not limited to the transforming growth factor beta (TGF-β), WNT, NOTCH, and PI3K-AKT pathways.^153,154^ Accumulating evidence has indicated that repurposed drugs may have a significant role in targeting these pivotal signaling pathways, and may, therefore, exhibit potential antitumor effects across diverse metastatic cancer models.

#### Mebendazole

Mebendazole (MBZ; 5-benzoyl-1H-benzimidazol-2-ylcarbamate) first described in 1968, was initially recognized as a broad-spectrum anthelmintic agent and was applied to humans in 1971.^155^ Fast forward two decades, and the focus on anthelmintics shifted towards their potential anticancer properties, primarily due to their interactions with microtubules.^156–159^ MBZ has been shown to potentially suppress tumor growth in various cancer cell lines and animal models through the inhibition of microtubule polymerization, a process that, when interrupted, can lead to the death of rapidly dividing cells. Significantly, the anticancer effects of MBZ extend to inhibiting the invasion and metastasis of malignant tumors. One study, in particular, highlighted the ability of MBZ to decrease integrin β4 expression and reduce CSC-like properties, which led to the shrinkage of primary tumors and a reduced risk of metastases, notably to the lungs and liver.^160^ MBZ has also been shown to inhibit tumor growth via the JAK2/STAT3/Bcl-2 signaling pathway, while other studies have indicated that MBZ might modulate cancer cell migration through the S1P/FAK/vimentin pathway.^161^ MBZ has also been found to restrict the migratory and invasive tendencies of glioblastoma cells, and concurrently modulate pivotal markers in the EMT, suggesting a potential role for MBZ in mitigating glioblastoma metastasis. In oral squamous cell carcinoma, MBZ was found to downregulate specific proteins and enzymes, including FAK, Rho-A, and Rac1 GTPase. Moreover, in the TGF-β-induced dysplastic oral keratinocyte (DOK) cell line, which models EMT, MBZ was shown to disrupt the cadherin equilibrium, further accentuating its potential as an anticancer agent.^162,163^ Recent anecdotal evidence from two case reports has further supported the possibility of MBZ being repurposed as an anticancer drug by documenting its success in managing metastatic patients.^164,165^ Together, these findings demonstrate the critical need to learn more about the therapeutic profile of MBZ to ensure its safety in oncological applications and determine its efficacy as a groundbreaking anticancer treatment.

### DNA damage response

The DNA damage response (DDR), which is activated in response to numerous DNA damage events including germline or somatic defects in DNA repair,^166^ oncogene-induced replication stress,^69^ flawed mitotic chromosome segregation,^167^ clashes between replication and transcription machinery^168^ or even as a consequence of genotoxic anticancer treatments,^169^ is a crucial hallmark of cancer. The DDR is instrumental not just during the onset and development of cancer, but also in its treatment. Although the majority of studies on DDR have focused on the role of poly (ADP-ribose) polymerase (PARP), the development of resistance to PARP inhibitors is becoming increasingly problematic in clinical settings since resistance exacerbates disease recurrence and worsens patient prognosis. Based on these findings, studies have shifted focus to other DDR targets, including ataxia-telangiectasia mutated kinase, ataxia telangiectasia and Rad3-related kinase, CHK1, and protein kinase, membrane-associated tyrosine/threonine 1. The use of repurposed drugs to target these entities presents a promising alternative strategy to combat genomic instability.

#### Genistein

Genistein (4’,5,7-trihydroxyisoflavone) is a naturally occurring isoflavone that is found in a vast range of foods.^170^ Notably, the median daily intake of isoflavones for adults in Japan and China is estimated to be between 25 and 50 mg, which is significantly higher than the intake levels of Western females. Interestingly, several epidemiological studies have reported that Asian countries exhibit significantly lower incidence rates of certain types of cancer, such as breast and prostate cancer, than Western countries.^171,172^ These discrepancies have fueled a surge of interest within the scientific community, which has prompted rigorous studies into the potential role of genistein in cancer prevention and suppression of tumor growth. The anticancer effects of genistein are thought to be intricately associated with its ability to modulate the mechanisms of DDR proteins, and thus position genistein as a central player in cancer research and prevention. Genistein has been identified as a potent inhibitor of DNA topoisomerase II, and is known for its ability to induce double-strand breaks in DNA by inhibiting the activity of this critical enzyme. One pivotal study demonstrated that genistein induced DNA damage in human lymphoblastoid TK6 cells. Furthermore, cells that lacked Ligase4 (a critical enzyme in the non-homologous end joining (NHEJ) pathway) displayed increased sensitivity to genistein. This heightened susceptibility was manifested through increased accumulation of γ‐H2AX foci and an increased number of chromosomal aberrations. These findings not only highlighted the collaborative roles of NHEJ and homologous recombination (HR) in the repair of genistein-induced DNA damage but also suggested that genistein has the potential to amplify the activity of drugs targeting DNA damage including inhibitors of the NHEJ and HR pathways. Thus, genistein may be a potential adjuvant in therapeutic strategies that exploit the DDR for cancer treatment.^173^ Similarly, Liu et al. found that genistein inhibited the phosphorylation of DNA-PKcs, subsequently suppressing the NHEJ repair pathway and delaying the HR repair process. In addition, they demonstrated that genistein sensitized DNA-PKcs-proficient glioblastoma cells to carbon ion radiotherapy. Genistein has also been shown to activate key proteins involved in the DDR, such as JNK and Ask1, which play crucial roles in various cellular processes, including DNA damage, caspase-3 activation, DNA fragmentation, and the downregulation of thioredoxin-1. Together, these studies highlight the multifaceted impact of genistein on cellular responses to DNA damage, as well as its potential to enhance the effectiveness of radiotherapy in specific cancer contexts. In addition, these findings provide valuable insights into the mechanisms underlying the anticancer properties of genistein and solidify its standing as a promising therapeutic agent.^174^ Finally, genistein has also been shown to reduce NNKAc-induced ROS and DNA damage through the activation of Nrf2, demonstrating its protective role against oxidative stress and DNA damage.^175^ To date, numerous clinical trials have been initiated to determine the therapeutic potential of genistein against various types of cancer, including NSCLC (NCT01628471), pancreatic cancer (NCT00376948), and breast cancer (NCT00769990). Predominantly, these studies have aimed to investigate the synergistic effects of combining genistein with radiotherapy and chemotherapy, thereby elucidating its potential as a chemopreventive agent, particularly when used in conjunction with other treatment modalities. Although initial findings have been promising, it is imperative to acknowledge that translating the therapeutic benefits of genistein from preclinical models to clinical application necessitates exhaustive and meticulous research to validate its efficacy and safety in human subjects.

### Targeting tumor-promoting inflammation

Inflammation significantly contributes to the progression of cancer through the generation of angiogenic factors and metastasis-associated proteins that can intensify or promote tumor invasion, migration, and malignancy via interactions with the TME.^176,177^ Consequently, using repurposed drugs to modulate inflammation has emerged as a potent anticancer strategy.

#### Celecoxib

Celecoxib, a selective cyclooxygenase-2 (COX-2) inhibitor, is conventionally prescribed for adult arthritis.^178^ However, recent studies have described the anticancer properties of celecoxib, which are mediated through the suppression of COX-2, a factor that is closely associated with cancer-related inflammation by promoting the synthesis of various prostaglandins, such as prostaglandin E2 (PGE2). Celecoxib has been shown to enhance the chemosensitivity of platinum-treated GC cells through the inhibition of prostaglandin-endoperoxide synthase 2 (PTGS2) and Bcl2 expression via the ERK1/2 and P38 signaling axis. PTGS2 has also been shown to reduce the cytotoxic effects of cisplatin (DDP) on GC cells through the PGE2/EP4/MAPKs (ERK1/2 and P38) axis.^179^ The significant role of inflammation in the malignant evolution of multiple cancers, mediated largely through the NF-κB signaling pathway, has been consistently emphasized in multiple studies.^180–182^ Celecoxib has been shown to enhance the efficacy of BRAF/MEKi treatments in melanoma through suppression of the NF-κB pathway. Since COX-2 promotes the resistance of melanoma cells to kinase inhibitors through the regulation of NF-κB -mediated inflammatory mediators, celecoxib can effectively counter the tumor-promoting actions of COX-2 by inhibiting its expression.^183^ Interestingly, Zhang et al. reported that celecoxib increased PTEN protein expression while inhibiting NF-κB and phosphatase of regenerating liver-3 expression levels in HCC-afflicted mouse livers.^184^ A compelling study by Guo et al. demonstrated that administration of celecoxib post-diagnosis led to better overall survival rates in cancer patients, particularly those exhibiting positive PTGS2 expression combined with a phosphatidylinositol-4,5-bisphosphate 3-kinase catalytic subunit alpha (PIK3CA) mutation.^185^ Overall, the momentum of clinical trials investigating the role of celecoxib in cancer therapy is intensifying. In particular, the potential synergistic combination of celecoxib with chemotherapy or immunotherapy could improve cancer treatment outcomes, and confirm the importance of modulating inflammation as a potential therapeutic strategy against cancer.

### Locking phenotypic plasticity

During organismal development, cells often undergo terminal differentiation as they are organized into specific tissues. Cellular differentiation typically leads to anti-proliferative outcomes, which form a vital component of the body’s defense against tumor formation. However, when the usually restricted capabilities for phenotypic plasticity become unrestrained, tumor cells might bypass terminal differentiation.^186^ There are three manifestations of phenotypic plasticity: dedifferentiation, blocked differentiation, and transdifferentiation, each representing a distinct disruption to cellular differentiation.^67^ From a conceptual standpoint, a tumor’s resistance to differentiation can be counteracted by increasing expression of developmental transcription factors like mothers against decapentaplegic homolog 4 (SMAD4) and homeobox A5.^187,188^ Specifically, SMAD4 can promote differentiation, thereby suppressing WNT-induced proliferation.^189^ Exploiting these mechanisms through drug repurposing might offer novel groundbreaking therapeutic approaches.

#### Metformin

Metformin, a well-established oral hypoglycemic agent from the biguanide class, has gained prominence over the years due to its cost-effectiveness and favorable safety profile.^190,191^ Interestingly, long-term use of metformin in type 2 diabetes patients has been linked to a decline in tumor incidence and a reduced rate of cancer-related deaths.^192–196^ Recent studies have indicated that metformin might exert direct anticancer effects on a range of tumor cells, including the elusive CSCs,^197,198^ thereby highlighting the multifaceted impact of metformin on cancer cells, as well as its potential to reduce phenotypic plasticity. Chang et al., for example, demonstrated that metformin reduced hepatocyte nuclear factor 4α (HNF4α) levels via activation of AMPKα, which subsequently modulated the WNT signaling pathway.^199^ A different study reported that metformin inhibited HNF4G activity via AMPK-driven phosphorylation and ubiquitin-mediated degradation, which suppressed the invasive abilities and metastasis of SMAD4-deficient pancreatic ductal adenocarcinoma cells.^200^ Intriguingly, metformin also appears to suppress the self-renewal capacities of CSCs, as evidenced by diminished expression of CSC markers (such as CD44 and CD133) and the growth of tumor spheroids through activation of AMP-activated protein kinase (AMPK) and inhibition of protein prenylation in the mevalonate pathway.^201^ Consistent with these findings, a meta-analysis revealed a 45% risk reduction in thyroid cancer among metformin users in Eastern countries, a phenomenon which was more evident in Asian populations than their Western counterparts.^202^ Another extensive meta-analysis ascertained a notably lower gynecological cancer occurrence in those undergoing metformin treatment compared to alternative therapies (gynecological cancer: HR = 0.60, 95% CI: 0.49–0.74; endometrial cancer: HR = 0.65, 95% CI: 0.50–0.85; ovarian cancer: HR = 0.47, 95% CI: 0.27–0.82).^203^ Thus, metformin has emerged as a paradigm of successful drug repurposing for oncological applications. Preclinical, epidemiological, and clinical insights have confirmed that metformin may act as a metabolic modulator by targeting various molecular pathways. Thus, metformin is a promising potent adjunct in anticancer regimens that could potentially synergize with chemotherapy, targeted agents, and immunomodulators.

### Suppressing nonmutational epigenetic regulation

Recent studies confirming the significance of non-mutational epigenetic regulation in oncology have introduced an intriguing perspective on genome reprogramming that appears to operate independent of mutations, thus emphasizing a role for mutation-free pathways in cancer evolution. Many of these mechanisms are deeply interconnected with microenvironmental cues that govern epigenetic reprogramming. A prime example is hypoxia, a common trait within tumors, which profoundly modifies the TME. A direct consequence of hypoxia is the diminished activity of ten eleven translocation (TET) demethylases, which leads to marked changes in the methylome, especially elevated methylation levels.^204^ In addition, metabolic shifts that occur within the TME also play pivotal roles. For example, acetyl-CoA, derived from butyrate, drives histone acetylation, which subsequently modulates gene expression by activating histone acetyltransferases and suppressing histone deacetylases (HDACs) in both acyl-dependent and -independent manners.^205^ Furthermore, mounting evidence has suggested that repurposed drugs that target the TME can produce potent antitumor effects. Thus, drug repurposing aimed at the TME is a promising approach for counteracting non-mutational epigenetic shifts.

#### Baicalein

The roots, seeds and bark of *Oroxylum indicum* (L.), a traditional herbal medicine in China, India and other countries, has been used to treat a wide range of ailments including dysentery, rheumatic discomfort, diarrhea, pharyngitis, and persistent coughs, as well as more severe respiratory conditions like bronchitis.^206^ Baicalein (BE), a prominent flavonoid derived from the roots of *O. indicum*, possesses anti-oxidant, anti-inflammatory, anti-hepatotoxic, anti-viral, and antitumor properties.^207,208^ More recently, a role for BE in non-mutational epigenetic regulation has been described. Specifically, BE has been shown to reverse hypoxia-induced resistance to tamoxifen (TAM) through the downregulation of HIF-1α levels in breast cancer cells.^209^ From a molecular perspective, the 6-phosphogluconate dehydrogenase-driven oxidative pentose phosphate pathway is thought to facilitate the reshaping of histone H3K9 and DNA methylation patterns during tumor progression. Such changes lead to upregulation of N-cadherin transcription, a hallmark of EMT, and subsequent promotion of N-cadherin-induced distant metastasis.^210^ Interestingly, the intriguing interactions between BE and HIF-1α, the glycolytic regulator hexokinase I, and other glycolysis-associated genes suggest a role for BE in controlling the glycolytic pathway and subsequently regulating the energy dynamics of gastrointestinal cancer cells.^211^ BE is a major component of PC-SPES, a herbal concoction enriched with *Scutellaria baicalensis*, which was meticulously formulated under strict quality controls, and investigated in a phase 1 clinical trial in 2008. The trial was aimed at hormone-refractory prostate cancer patients and revealed encouraging outcomes both in terms of therapeutic efficacy and safety.^212^ Despite these promising results, further studies are required to elucidate the intricate mechanisms and better understand the broader implications of using BE as a potential therapeutic agent for the treatment of cancer.

### Decreasing polymorphic microbiomes

The human microbiota predominantly colonizes epithelial surfaces, with the most significant concentration found within the gastrointestinal tract.^213,214^ Gut microbiota orchestrate a range of vital physiological functions, from nutrient and drug metabolism and vitamin synthesis to immune regulation and preservation of gastrointestinal structure.^215,216^ Intriguingly, although many of these microorganisms play benign or even beneficial roles, some can also contribute to disease progression, including cancers. An imbalance in microbial ecology, characterized by a shift in microbiota composition and disrupted homeostasis, is often observed during tumor development.^217,218^ For example, the Fusobacterium adhesin A antigen found in *Fusobacterium nucleatum* (*F. nucleatum*) has been associated with the promotion of CRC progression via the E-cadherin/WNT-β-catenin signaling pathway.^219^ While some microorganisms affect tumorigenesis directly, others enhance antitumor immune responses, effectively serving as immune adjuvants. This interaction between microbes and immunity in the context of cancer has been coined the ‘immune-oncology-microbiome axis’.^214^ Emerging evidence has highlighted the intricate relationship between the gut microbiota and conventional anticancer treatments, such as chemotherapy, radiotherapy, targeted therapy, and immunotherapy. Thus, a thorough understanding of the multifaceted biological roles of the gut microbiome, along with the associated molecular pathways, is imperative. Such insights would allow for the discovery of new targets for cancer interventions and subsequent clinical evaluations.^220^

#### Inulin

Inulin (C_17_H_19_NO_3_), a quintessential soluble dietary fiber predominantly sourced from plants such as chicory, ginger, garlic, onion, and asparagus,^221^ has emerged as a multifaceted ingredient in the culinary and pharmaceutical sectors. Known for its versatility, inulin functions as a prebiotic, a salubrious substitute for fats and sugars, a texture enhancer, and a cornerstone in the formulation of functional foods.^222^ Contemporary research has highlighted the potential efficacy of inulin in oncological interventions, particularly due to its capacity to modulate the polymorphic microbiome of the gut. An exemplary application has been observed in the amalgamation of inulin, cellulose, and their derivatives in preventing liver metastasis associated with CRC, primarily through the modulation of gut microbiota. Further scrutiny has revealed that inulin’s putative anticancer properties may emanate from its influential role in reshaping the composition of the intestinal microbiota. This involves not only the synthesis of short-chain fatty acids but also the nuanced regulation of the gut microbiota’s dynamics and their metabolic offshoots, thereby offering a holistic strategy for cancer prophylaxis.^223^ Intriguingly, experimental studies on animal models have revealed that oral administration of inulin gel significantly amplifies the efficacy of immune checkpoint therapy while maintaining an admirable safety profile. This gel has the propensity to reformulate the composition of the gut microbiota and its metabolic outputs, consequently invigorating the immune system and potentiating an antitumor-immune response.^224^ Moreover, inulin has shown a pronounced ability to inhibit tumor proliferation and extend the latency phase of oncogenesis. In a landmark study by Wu et al., rodents fed an inulin-enriched diet exhibited a markedly diverse and robust gut microbiome compared to their counterparts. The inclusion of inulin in the diet resulted in a significant increase in plasma propionate levels and a concomitant decline in the expression of pivotal epigenetic regulatory proteins such as HDAC2, HDAC8, and DNA methyltransferase 3b. Concurrently, there was a discernible decrease in the expression of proteins pivotal to tumor cell proliferation and survival, such as Akt, phospho-PI3K, and NF-kB. This study highlights the potential of dietary inulin as an avant-garde tactic in the preventative arsenal against breast cancer, potentially leveraging epigenetic mechanisms to manifest its prophylactic effects.^225^ Furthermore, inulin has been credited with fostering the proliferation of beneficial gut flora while impeding the growth of deleterious bacteria in an animal model harboring the murine pks + E. coli strain NC101. This prebiotic agent not only recalibrates the equilibrium of the gut microbiota but also augments the functionality of intestinal immune cells, thereby magnifying the efficacy of immune responses. Inulin has also been shown to modulate the expression of genes associated with colon cancer, effectively curbing tumor growth and metastasis.^226^ In summary, inulin possesses a range of physiological benefits, with a particularly pronounced impact on cancer therapeutics. Nevertheless, the integration of inulin into clinical practice necessitates further human trials to affirm its safety and to elucidate the nature of its decomposition products within the human body.

### Targeting senescent cells

The four hallmarks of cellular senescence include: (i) a consistent, often irrevocable, cell-cycle arrest; (ii) the emergence of a senescence-associated secretory phenotype (SASP); (iii) macromolecular damage; and (iv) metabolic shifts.^227^ Serving as a countermeasure to programmed cell death, the primary function of cellular senescence is to remove damaged cells, including those predisposed to malignant transformation, thereby providing a safeguard against cancer.^228^ As such, the initiation of cellular senescence can pose a barrier to tumor development, presenting itself as a potentially favorable outcome for anticancer therapies. However, paradoxically, over the past decade, senescent cells have been shown to promote tumor growth and malignancy through a variety of mechanisms under certain circumstances.^229–231^ For example, DOX-induced systemic senescence was found to promote metastasis in an orthotopic mouse model of breast cancer. However, these harmful effects were mitigated either through genetic manipulation or pharmacological removal of senescent cells.^232^ Pioneering research has shown that senescent cells possess the capability to initiate malignancy in benign cells both in culture and in animal models. Moreover, in immunocompromised mice, senescent cells were shown to promote the growth of fully malignant breast cancer cells.^233,234^ Given these insights, the ability of repurposed drugs to act as both inducers of senescence and therapeutic agents targeting senescence has been acknowledged. Thus, the development of senescence-based therapy using repurposed drugs is an innovative therapeutic approach.

#### Quercetin

Quercetin, a potent flavonoid, is abundant in various plants, fruits, and vegetables, predominantly in glycoside forms found in onions, apples, blueberries, and broccoli.^235^ Its anti-inflammatory and antioxidant properties, as well as its ability to modulate the TME, have led to its inclusion in functional foods as a commercial dietary supplement.^235^ Recent studies have delved deep into its myriad of biological functions, particularly highlighting its anti-inflammatory, antioxidant, and anticancer properties.^236^ One of the most promising avenues for quercetin lies in its potential as a senotherapeutic agent. For example, in T24 bladder cancer cells, nuclear morphology analysis (NMA) revealed that quercetin treatment led to a marked increase in the percentage of cell nuclei during cellular senescence.^237^ Similarly, in Colo-320 and Colo-741 cells, quercetin treatment led to increased expression of several senescence markers including lamin B1, p16, and cyclin B1.^238^ Furthermore, quercetin was found to promote senescence in glioma cells by inhibiting the activity of HDACs. Moreover, in HepG2 liver cancer cells, quercetin was shown to reactivate P53, thereby inhibiting RNA degradation and protein ubiquitination, leading to the upregulation of P21 expression and concurrent downregulation of cyclin D1, a crucial player in cell cycle arrest.^239^ Thus, quercetin stands out as a potent tool to induce senescence in cancer cells. In the burgeoning field of senolytic treatments, combining quercetin with other agents has also shown promise in efficiently targeting senescent cells. Notably, a study by Zhu et al. revealed that quercetin (10 μM) could induce cell death in mouse bone marrow-derived senescent MSCs and radiation-induced senescent endothelial cells. In addition, pre-treatment with quercetin protected against DOX-induced normal cell senescence by reducing the number of senescent cells and suppressing the release of SASP factors.^240^ The ability of cancer cells to exploit senescence as a defense mechanism against therapies necessitates innovative approaches. Repurposing quercetin as an oncological drug has emerged as a strategic solution, as evidenced by a Phase II clinical trial that combined dasatinib and quercetin for the treatment of head and neck squamous cell carcinoma (NCT05724329). However, the broader application of quercetin in oncology has several challenges, and issues such as its limited bioavailability, instability, and lack of precise tumor targeting need to be addressed. Thus, novel strategies that augment the bioavailability of quercetin using lipid nanoparticles and chitosan nanoparticles have been developed. For example, a recent Phase II clinical trial assessed the therapeutic efficacy of both quercetin and its nanoparticle variant against oral squamous cell carcinoma cell lines (NCT05456022). In summary, the potential repurposing of quercetin is full of promise, highlighting the need for continued exploration and clinical validation.

### Summary

During the early stages of cancer management, various chemotherapy agents and targeted therapies often yield promising results. However, as treatment progresses, tumors display remarkable resilience, developing adaptive resistance through mutations in treatment targets or by activating alternative signaling pathways. This adaptability often undermines or even neutralizes the effectiveness of therapeutic interventions. Furthermore, activation of survival pathways or suppression of death signals further promotes resistance. Current research has indicated that repurposed drugs, which target a range of malignant features, might enhance the potency of existing anticancer agents. In this section, we described repurposed drugs that target multiple malignancies in cancer, and revealed their potential for combination therapies, including immunotherapy, chemotherapy, and targeted treatments. While only a few repurposed drugs have been recognized for their direct anticancer effects, their multifaceted therapeutic targets are noteworthy. Looking ahead, integrating these drugs either as supportive agents in cancer care or in tandem with established anticancer agents may set the stage for more sustainable and effective cancer treatments. Such an approach has the potential to increase the impact of current cancer therapeutics.

## Drug repurposing: candidates for TME-targeting therapy

**Fig. 4:**
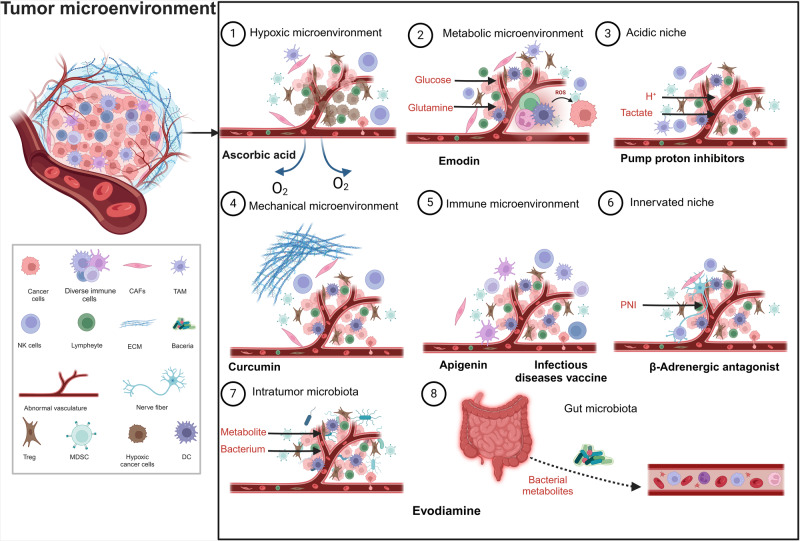
Classification of the tumor microenvironment. Underlying mechanisms of repurposed drugs targeting the specialized tumor microenvironments (TMEs). The TME can be divided into seven specialized microenvironments: hypoxic niche, immune microenvironment, metabolic microenvironment, acidic niche, innervated niche, mechanical microenvironment, and microbial microenvironment. Repurposed drugs with multi-targeted effects may reverse the effects of tumor-promoting microenvironments. TAM tumor-associated macrophage, CAF cancer-associated fibroblast, MDSC myeloid-derived suppressor cell, PNI perineural invasion, NK natural killer. This figure was created with Biorender.com

The TME contains various immune cells, such as helper T (Th) cells,^241^ Tregs,^242^ dendritic cells,^243^ TAMs,^244^ and mesenchymal stem cells (MSCs),^245^ as well as fibroblastic stromal cells, including cancer-associated fibroblasts (CAFs), which surround tumor cells and are sustained by adjacent blood vessels. By secreting a range of molecules, these cells can either directly stimulate cancer cell proliferation or modify the molecules within their surroundings, which promotes tumor growth.^246,247^ The TME supports the survival and migration of cancer cells throughout the organism in response to internal or external stimuli, including treatments.^248^ Thus, an accurate and detailed understanding of the TME and other specialized TMEs will be beneficial in the development of potential cancer therapies.^249^ With respect to the different hallmarks of the TME, in the current section we will elaborate on the tumor immune microenvironment (TIME), metabolism microenvironment, hypoxic microenvironment, acidic niche, mechanical microenvironment, and innerved niche (Fig. 4), since they represent the critical aspects of the TME. Gut microbiota and their metabolites also play an important role in tumorigenesis. Various metabolic by-products of bacteria such as lactic acid,^250^ adenosine, nitric oxide (NO),^251^ potassium ions (K^+^),^252^ and ROS^253^ accumulate in the microenvironment, resulting in abnormal pH and oxygen levels, which promote cancer growth.^246^ To date, multiple studies have demonstrated that non-oncological repurposed drugs can produce unique antitumor effects by targeting one or more of the specialized microenvironments outlined above.

### Immune microenvironment

Targeting and reversing the immunosuppressive attributes of the TME is critical for exploiting the TIME for therapeutic gain. Numerous studies have reported that immunosuppressive cells, ranging from Tregs and B cells, are recruited during tumor progression.^254,255^ These cells have been found to suppress the immune system by inhibiting both the trafficking and functionality of T cells through direct and indirect mechanisms.^256^ Moreover, other components of the TIME, such as TAMs, monocytes, and granulocytes, have also been reported to inhibit the antitumor activity of T cells and NK cells via various mechanisms, thereby leading to resistance to immunotherapy.^244,257^ Given this highly intricate milieu, a synergistic approach integrating ICIs with therapies that specifically target the TIME such as cytokine therapy, oncolytic viruses, and anti-angiogenic treatments could potentially increase antitumor immune responses. Concurrently, emerging research has explored the repurposing of traditional drugs to target pivotal facets of the TIME, such as CAFs and the extracellular matrix (ECM), offering innovative avenues to strengthen the potency of immunotherapeutic interventions.

#### Apigenin

Apigenin is a flavonoid ubiquitously found in numerous fruits and vegetables. Due to its anti-inflammatory, antioxidant, antitumor, anti-microbial, anti-viral, and cardiovascular protective properties, interest in apigenin has recently increased in the field of immune microenvironment research.^258^ Recent studies have revealed the potential of apigenin to act as an immunomodulator, positioning it as a promising candidate for cancer therapy repurposing. For example, in specific breast cancer (MDA‐MB‐468, SK‐BR‐3, and 4T1) and melanoma (A375, A2058, and RPMI‐7951) cell lines, apigenin has shown efficacy in negating the upregulation of PD‐L1, an effect induced by IFN‐γ. This capacity to modulate PD‐L1 has been further confirmed in co-culture settings with Jurkat T cells, where apigenin was shown to not only increase T lymphocyte proliferation, but also promote their apoptosis in breast cancer (MDA‐MB‐468) and melanoma (A375) cells.^259,260^ These findings highlight the intertwined relationship between T cell activation, apigenin-dependent PD-L1 regulation, and subsequent cancer cell demise. Apigenin has also been shown to modulate the transcription of SHIP-155 through the suppression of miRNA-1, demonstrating its pivotal role in orchestrating the antitumor immune dynamics within both the bone marrow and the TME of mice with pancreatic cancer.^261^ To date, only one clinical trial (NCT00609310) has examined the clinical application of apigenin in CRC. The lack of clinical trials may be due to its limited bioavailability and stability. Nonetheless, the development of advanced delivery mechanisms designed to overcome these obstacles may broaden the clinical application of apigenin in the future.

#### Infectious disease vaccines

Many non-oncological drugs and vaccines have emerged as potential candidates for repurposing in cancer treatment, with some already incorporated into clinical practice. The FDA-approved anticancer infectious disease vaccines currently in use include Bacillus Calmette-Guerin (BCG; bacterial-based), Talimogene laherparepvec (TVEC; an oncolytic virus), and Provenge® (Sipuleucel T; a dendritic cell-based vaccine).^262^ Thus, vaccines pose a promising approach to target the TIME in cancer treatment. Yao et al. reported that BCG heightened the cytotoxicity of GC cells by inducing lymphocytes to secrete IFN-g. In addition, BCG was found to augment the phagocytic capacity of human THP-1 monocytes/macrophages against *H. pylori*, by increasing the expression of the surface integrins CD11b, CD11d and CD18, as well as the membrane and soluble lipopolysaccharide receptors CD14 and sCD14, ultimately reducing the incidence of GC.^263^ Another study reported that intratumoral delivery of the prophylactic yellow fever vaccine (live 17D) into mice stimulated immune-mediated antitumor effects including the orchestration of cytotoxic T lymphocytes and suppression of Tregs.^264^ Intriguingly, Tai et al. found that administering a single dose of the influenza vaccine intramuscularly a day before surgery optimally activated NK cells in specific murine models.^265^ Based on these findings, a phase 1 clinical trial (NCT02998736) was initiated to evaluate the safety and NK cell killing activity of perioperative administration of the intramuscular influenza vaccine. The trial was carried out in conjunction with the phosphodiesterase-5 inhibitor, tadalafil, to counteract myeloid-derived suppressor cell (MDSC) inhibition of NK cells in patients undergoing abdominal cancer surgery.^266^ Currently, multiple infectious disease vaccines are the focus of oncology-based preclinical and clinical trials. Repurposing such vaccines to target the TIME could be a cost-efficient approach to expand the therapeutic alternatives available to patients.

### Metabolism microenvironment

As previously discussed, the reprogramming of metabolism is regarded as one of the hallmarks of cancer. The reconfiguration of catabolic and anabolic processes in cancer cells contributes significantly to tumorigenesis and disease progression.^99^ In cancer cells, tailored modification of metabolic flux across diverse pathways caters to the heightened energy and synthesis demands intrinsic to tumor proliferation.^267^ More recently, it has become apparent that not only the metabolism of cancer cells but also that of stromal and immune cells within the TME plays a pivotal role in tumor maintenance. Cells that support tumor maintenance include endothelial cells (both vascular and lymphatic) and their affiliated pericytes, CAFs, and a range of immune cells including tumor-infiltrating lymphocytes (TILs) such as T cells, B cells, NK cells, TAMs, and mast cells.^268,269^ Endothelial cells are among the most studied stromal cells within the TME. Lactate uptake by endothelial cells has been shown to induce angiogenesis by increasing the expression of IL-8, VEGF, VEGFR2 and basic fibroblast growth factor (bFGF), as well as the phosphorylation of AKT, thereby promoting tumor growth. Moreover, CAFs reportedly secrete lactate, which tumor cells can use as a metabolic fuel by incorporating it into oxidative phosphorylation (OXPHOS) in the mitochondria, thereby supporting their high proliferation rates. This phenomenon is known as the “reverse Warburg effect”.^270^ Tumor metabolic reprogramming also plays a pivotal role in shaping the immune response, specifically antitumor immunity. For example, high expression of hexokinase 2 (HK2) in tumor cells can suppress transcription of the gene encoding IFN-*γ*, thereby contributing to immune response evasion.^271^ This concept of “oncometabolites” has ushered in a new era for cancer treatment. In terms of potential therapies, targeting the metabolic characteristics of different cells within the TME will enable the development of novel drug-repurposing strategies to combat cancer, which can be used in combination with traditional metabolic approaches.

#### Emodin

Emodin, a quintessential anthraquinone derivative from traditional Chinese herbs, has been increasingly recognized for its diverse biological activities, which include anticancer, antibacterial, liver-protective, and anti-inflammatory properties.^272,273^ More recently, the role of emodin in metabolic reprogramming has become an area of interest, and its role as a promising metabolic inhibitor has expanded its potential applications for cancer treatment. Emodin has been shown to suppress cholesterol biosynthesis in human HCC cells by attenuating the transcriptional activity of SREBP2 and inhibiting AKT signaling.^274^ Similarly, other studies have demonstrated that emodin regulates SREBP1 leading to a reduction in triglyceride levels and fatty acid desaturation, and ultimately inducing apoptosis in liver cancer cells.^275^ Furthermore, Xing et al. demonstrated that the cytotoxic effects of emodin on HepG2 cells were associated with disruptions in metabolic homeostasis including oxidative stress and imbalances in amino acids.^276^ Moreover, in addition to its lipid metabolism modulatory functions, emodin has also been shown to induce apoptosis in cervical cancer cells through the suppression of HPV E6/E7 expression and modulation of glucose metabolism.^277^ Despite these encouraging results, few clinical trials have focused on emodin due to its limited bioavailability. In the future, greater emphasis needs to be placed on exploring the clinical implications of emodin, as well as its therapeutic applications.

### Mechanical microenvironment

During tumor development and progression, dynamic and heterogeneous interactions between tumor cells and the surrounding microenvironment occur, such that cells within the TME are subjected to both external and internal forces. In response to these mechanical pressures, including solid stress, shear stress, increased matrix stiffness, and topological changes, cells experience mechanosensing and mechanotransduction. These processes promote tumor progression by influencing phenomena such as the EMT and enhancing cell survival through autophagy.^278,279^ The formation of a mechanical microenvironment primarily depends on collagen and fibrin, integrins and fibroblasts. These components play important roles in regulating cell adhesion,^280^ morphology,^281^ motility, proliferation, differentiation, and migration.^282^ CAFs reportedly secrete matrix metalloproteinases (MMPs), including MMP-2, MMP-3, and MMP-9, or activate Yes-associated protein to promote ECM degradation and remodeling, EMT, and CSC stemness.^283–287^ Many studies have focused on the cellular proteins involved in mechanosensing, such as integrins and focal adhesion proteins, and their associated molecular mechanisms including cytoskeleton remodeling, integrin signaling, Rho signaling, and Hippo signaling.^281^ Consequently, treatments targeting the mechanical TME represent an emerging strategy in targeted therapy.

#### Curcumin

Curcumin, a vibrant yellow polyphenol derived from the turmeric plant (*Curcuma longa*), boasts an impressive range of bioactive properties and has been used to treat dermatological conditions.^288,289^ Interestingly, curcumin has displayed antitumor properties both in vitro and in vivo, and has been shown to act through multiple cellular pathways. It is one of the few compounds that has progressed to clinical trials.^290^ Of note, curcumin has recently been found to exert antitumor effects by targeting the mechanical microenvironment. Jalilian et al. showed that curcumin attenuated expression of the genes α-SMA and COX-2 and the production of PGE2 in CAFs. This study further demonstrated that curcumin effectively altered the pro-tumor characteristics of CAFs by suppressing PGE2 gene expression, leading to upregulation of the T-bet gene and increased production of interferon-gamma, which collectively contribute to a marked decrease in inflammation within the TME. As a result, these changes significantly enhance the antitumor capabilities of immune cells.^291^ The potential of curcumin to reduce metastasis of breast cancer is also evident in its inhibition of CXCL12/CXCR4 axis-mediated activation of the differentiation of adipose-derived MSCs (ADMSCs) into CAFs.^292^ In addition, Mao et al. demonstrated that curcumin suppressed proliferation of LGR5(+) colorectal CSCs by not only promoting autophagy but also through transcriptional repression of the oncogenic transcription factor activating enhancer-binding protein 2A (TFAP2A)-regulated ECM pathway.^293^ Although the antitumor properties of curcumin have been confirmed by multiple pre-clinical and clinical studies, its transition into mainstream cancer treatment has been limited by its subpar bioavailability. Furthermore, clinical investigations on curcumin have often involved small cohorts, which can lead to clinical variability. As a result, more expansive and well-structured clinical trials are required in the future to confirm that curcumin is a potential therapeutic agent for the treatment of cancer.

### Hypoxic microenvironment

Hypoxia, a deficiency in the amount of oxygen reaching the tissues, is a defining feature of the TME and acts as an important negative prognostic indicator across numerous solid tumors.^294^ Hypoxia occurs when there is an imbalance between the oxygen supply and oxygen requirements of cancerous and stromal cells, and often involves a malfunctioning microvascular system. This oxygen-deprived environment plays a pivotal role in shaping the biological characteristics and aggressive nature of cancer cells.^295^ HIF-1α serves as a primary marker of the hypoxic environment. Under low-oxygen conditions, elevated HIF-1α activity leads to the upregulation of Snail and Twist, transcriptional regulators that suppress E-cadherin expression resulting in EMT. The adverse effect of hypoxia is evident in multiple types of cancer, while its intricate role in modulating the efficacy of chemotherapy, radiotherapy, and immunotherapy cannot be overstated. Notably, Wenger et al. examined the impact of hypoxia on mouse embryonic fibroblast proliferation, and found that the suppressive effects of agents like carboplatin and etoposide on cell growth were significantly increased when HIF-1α was deactivated.^296^ Given these insights, targeting the hypoxic TME via drug repurposing may be beneficial in the development of oncological agents.

#### Ascorbic acid

Ascorbic acid, commonly known as vitamin C, is an essential water-soluble vitamin that humans are unable to synthesize endogenously, and must therefore obtain from dietary sources.^297,298^ As an antioxidant, ascorbic acid protects against free radicals, and is involved in several vital physiological functions including collagen formation, wound healing, tissue repair, and maintaining the health of bones, cartilage, and teeth.^299^ More recently, groundbreaking preclinical studies have shed light on the novel roles of vitamin C as a supplementary agent in a range of innovative cancer treatment approaches that involve epigenetics, immunomodulation, and selective cytotoxicity. Recent studies have established a compelling link between the anticancer properties of ascorbic acid and the hypoxic microenvironment within tumors, which has placed significant emphasis on the role of HIF-1α. For example, in human endometrial tumors, elevated levels of ascorbic acid within the tumor have been shown to correlate with decreased expression of HIF-1α, VEGF, and GLUT1 proteins, leading to a marked reduction in malignancy.^300^ Furthermore, ascorbic acid has been shown to induce a dose-dependent decrease in the expression of HIF-1α and GLUT1, both identified as downstream targets of HIF-1, in thyroid cancer cells in vitro. Similarly, low concentrations of ascorbic acid (25 mM) have been shown to suppress HIF-1α expression in hypoxic conditions, subsequently impeding tumor growth.^301^ Moreover, in an in vivo subcutaneous lung tumor transplantation model in rats, intraperitoneal injections of ascorbic acid (1 g/kg) were found to reduce HIF-1α expression within the tumor, while simultaneously suppressing tumor growth and reducing vascular density.^302^ Furthermore, a retrospective analysis involving human patient tumor samples paired with controls for endometrial cancer, RCC, and CRC revealed that tumors exhibiting the highest HIF-1 activity were generally associated with patients who had a notable deficiency in ascorbic acid.^303^ Thus, the vast amount of preclinical and clinical data currently available indicates that ascorbic acid may be a promising agent for therapeutic repurposing, particularly with respect to its applications in targeted therapy and dietary treatment.

### Acidic niche

The typical pH of the interstitial space in solid tumors ranges from 6.4 to 7, which is more acidic than that of normal tissues, which maintain a pH between 7.3 and 7.4.^304^ During acidic selection, cancer cells acquire enhanced acid extrusion capacity and other properties that confer a high degree of fitness in evolutionary terms, leading to an increased propensity for proliferation, survival, and invasiveness.^305^ For example, exposure of melanoma cells to extracellular acidosis for 24 hours led to increased expression of EMT markers and enhanced in vitro invasiveness.^306^ In addition, the functionality of antitumor agents, such as T cells and NK cells, is often reduced under acidic conditions. In contrast, the actions of tumor-promoting cells such as MDSCs and Tregs are often increased under acidic conditions, leading to enhanced tumor growth and suppression of antitumor immune responses. Notably, in one study, a pH of 6.5 was found to reversibly impair T cell function, characterized by a reduced expression of T cell receptor components in melanoma patients.^307^ Thus, drug repurposing aimed at neutralizing acidic niches might offer a viable approach to stabilize pH levels within the TME.

#### Pump proton inhibitors

Proton pump inhibitors (PPIs) are the preferred drugs for inhibiting gastric acid secretion. They are commonly prescribed to treat conditions like peptic ulcers, gastroesophageal reflux disease, Zollinger-Ellison syndrome, and upper gastrointestinal bleeding.^308,309^ Over the years, PPIs have been administered to billions of people worldwide and have shown a remarkable safety profile even at high dosages. The ability of PPIs to counteract tumor acidity and reduce acid-induced chemoresistance has been the focus of recent studies. Multiple reports have demonstrated that PPIs modulate tumor acidification and renew chemosensitivity in resistant cancer cells both in vitro and in vivo.^310^ PPIs have been shown to reduce V-ATPase activity in the acidic niche of cancer cells, thereby disrupting proton transport, and subsequently shifting the pH balance within these cells resulting in increased intracellular concentrations of cytotoxic drugs.^311^ Similar findings have been reported in other in vitro studies, indicating that potential downstream effectors, such as the dephosphorylation of low-density lipoprotein receptor-related protein 6 and the inhibition of WNT/β-catenin^312^ or PI3K/AKT/mTOR/HIF-1α signaling pathways,^313^ follow the inhibition of V-type H^+^ ATPase by PPIs in GC cells. PPIs have also been found to counteract the effects of an acidic microenvironment on the ability of tumor cells to evade immune surveillance. For example, pantoprazole reportedly increased the number of TAMs in the TME, as well as enhanced CD11c expression and phagocytosis, and influenced macrophage shape.^314^ Interestingly, a recent meta-analysis suggested a heightened risk of cancer in PPI users versus non-users.^315^ This might be due to the inhibition of H^+^/K^+^ ATPase by PPIs in parietal cells, which leads to increased release of gastrin from G-cells.^316^ Gastrin has long been suspected to be a potential risk factor for GC. However, this analysis did not stratify the risk of GC by dose due to a lack of data. Given the historical association between gastrin and potential risks of GC, this correlation warrants closer examination, especially with respect to dosage. Moreover, a study of 6754 breast cancer patients revealed that the use of PPIs not only markedly improved their overall survival rates but also reduced the risk of disease recurrence.^317^ Notably, these PPIs also mitigated resistance commonly seen with conventional chemotherapy drugs and radiation treatments. Given these findings, PPIs have emerged as a promising adjunctive treatment strategy, especially when combined with other therapeutic drugs. Various PPIs are currently being utilized in the treatment of different cancers, including liver and breast cancer. As we look to the future, there is a compelling case for more clinical trials to accelerate the wider adoption of PPIs in cancer therapy.

### Innervated niche

The role of the nervous system in the development of solid tumors has often been underestimated. Although the potential significance of the peripheral nervous system in oncogenesis was suggested by pioneers in the field last century, the functional contribution of nerves to cancer development and modulation of the TME have remained under-explored for a long time.^318^ More recently, groundbreaking research has revealed that tumors can actively recruit nerves into the TME, which can then directly promote tumor growth—a phenomenon known as “tumor innervation”.^319,320^ These findings have highlighted novel perspectives on tumor–nerve interactions. Emerging evidence has suggested that certain neurochemicals, such as dopamine, catecholamines, and acetylcholine, have direct roles in tumor initiation and progression. For example, β-adrenergic signaling has been implicated in promoting EMT and increasing the metastatic potential in cancer cells via the VEGF/MMP and STAT3/ERK/MAPK pathways.^321^ Furthermore, Sloan et al. found that both stress-induced and pharmacological β-adrenergic stimulation promoted macrophage migration into tumor tissue in a mouse model of breast cancer. These macrophages then adopted an immunosuppressive M2 phenotype, leading to increased production of TGF-β, VEGF, and MMP-9, which in turn promoted angiogenesis and metastasis.^322^ Thus, a deeper understanding of the nerve-rich regions within tumors could be beneficial for the development of innovative cancer therapeutic strategies.

### β-adrenergic antagonists

β-adrenergic antagonists, commonly known as β-blockers, are primarily used to manage cardiovascular conditions such as hypertension and coronary artery disease, and act by inhibiting β-adrenergic receptors (β-ARs) within the adrenergic system.^323–325^ Recent studies have suggested that the antitumor effects of β-blockers might be due to disruption of the innervated niche. Catecholamines, for example, have been shown to activate macrophages via adrenergic signaling, leading to a shift towards the M2-polarized phenotype and increased VEGF production, which ultimately promotes tumor angiogenesis.^326,327^ This catecholamine-driven effect was found to be mitigated by the β-AR antagonist, propranolol. Furthermore, elevated catecholamine levels, often present during depressive episodes, have been shown to interact with β2-ARs on GC cells, thus promoting expression of metastasis-associated in colon cancer 1 (MACC1). MACC1 then binds to synaptophysin to preserve the neuroendocrine phenotype, which is associated with increased invasiveness and the metastatic potential of GC. This malignant transformation induced by catecholamines was effectively blocked by the β2-AR antagonist, ICI-118,551. In addition, Shi et al. demonstrated that ICI-118,551 could reverse catecholamine-induced resistance to trastuzumab in cancer cells, thereby improving the efficacy of targeted cancer treatments.^328^ In conclusion, β-adrenergic antagonists, repurposed as anticancer drugs, might be suitable candidates for combination therapies targeting disparate neoplastic processes. To date, most of the clinical trials on β-adrenergic antagonists have a relatively small sample size, or are still underway, with results yet to be published. Thus, there remains a critical need for extensive randomized controlled clinical trials with a specific focus on β-adrenergic antagonists.

### Microbial microenvironment

The microbial microenvironment is primarily comprised of intestinal and intra-tumoral microorganisms and their metabolites, which possess the ability to either facilitate or inhibit tumor progression and impact the effectiveness and toxicity of cancer treatments.^329–332^ For example, beneficial gut microbes have been shown to improve the antitumor effects of certain therapies such as ICIs by modulating the immune response.^333–335^ In contrast, other microbes can promote tumor initiation and progression directly by generating toxic or tumorigenic products or indirectly by shaping a pro- or anti-inflammatory microenvironment. Parhi et al. found that *F. nucleatum* accumulated specifically within tumors and reduced the number of tumor-infiltrating CD4^+^ and CD8^+^ T cells, indicating that *F. nucleatum*-mediated changes in the TIME contributed significantly to tumor growth.^336^ In addition, the *F. nucleatum* DNA load in MSI-high CRC tumors was found to be inversely correlated with the density of tumor-infiltrating FOXP3^+^ T cells, and positively correlated with the ratio of M2-like TAMs to total TAMs, suggesting that *F. nucleatum* primarily promotes tumor progression through the proliferation of suppressive immune cells.^337^ Therefore, studies into the causal relationship between the tumor microbial microenvironment and the overall TME may provide novel insights that contribute to the development of new drug research within the era of precision medicine.

#### Evodiamine

Evodiamine (EVO) is a natural alkaloid compound derived from the fruit of *Evodiae fructus*, a member of the *Rutaceae* family, that is primarily used for the treatment of various ailments such as vomiting, diarrhea, headaches, and challenging menstrual cycles.^338^ Beyond its traditional medicinal applications, EVO is also a popular dietary supplement.^339,340^ Recent studies have revealed a role for EVO in targeting the microbial microenvironment, which may provide potential anticancer benefits in a spectrum of malignancies. For example, Yang et al. found that EVO impeded the growth of *H. pylori* reference strains and clinical isolates, as well as reduced the translocation of CagA and VacA proteins into AGS GC cells.^341^ EVO appears to exert its protective effects against CRC through modulation of the gut microbiota and reduction of intestinal inflammation, potentially through inhibition of the IL6/STAT3/P65 signaling pathway.^342^ In addition to its direct anticancer effects, EVO has also been shown to temper inflammatory reactions and tumor-associated immune responses. Further analysis has revealed that EVO augments the presence of bacteria that produce short-chain fatty acids while reducing the number of pro-inflammatory bacteria.^343^ Such modulations affect microbiota metabolism through regulation of the tryptophan pathways. However, few clinical studies have focused on EVO, despite these promising preclinical insights and the substantial evidence outlining the safe dosages and pharmacological implications for tumor management. Thus, it is likely that EVO-infused medications may become integral in preventive and therapeutic healthcare strategies in the future.

### Summary

The TME is a dynamic and multifaceted milieu that consists of multiple cellular entities and molecular constituents that often dictate the behavior of nearby cancer cells. Given its intricate nature and profound influence on cancer progression, the TME has attracted interest recently as a potential therapeutic target.^344^ In this section, we evaluated the anticancer activity of repurposed drugs from multiple perspectives including the TIME, metabolism microenvironment, mechanical microenvironment, hypoxic microenvironment, acidic niche, innervated niche, and microbial microenvironment. Presently, there is a lack of clinical trial evidence describing the efficacies of these repurposed agents in modulating the TME, which probably reflects our nascent understanding of the unique anticancer mechanisms each drug embodies. Thus, it is crucial to understand that cancer is not a monolithic entity, but a continuum, evolving through multiple stages. Accordingly, repurposed drugs might manifest distinct effects contingent on the phase of the disease. In the future, it is necessary to conduct more nuanced preclinical studies and tailored clinical trials that identify the most efficacious therapeutic strategies. The re-emergence of repurposed drugs for oncological purposes signals a renaissance in their applicability, and paves the way for future studies aiming to harness their full potential against both the TME and the malignancies they harbor.

## Drug repurposing: candidates for nanomaterial-based therapy

**Fig. 5:**
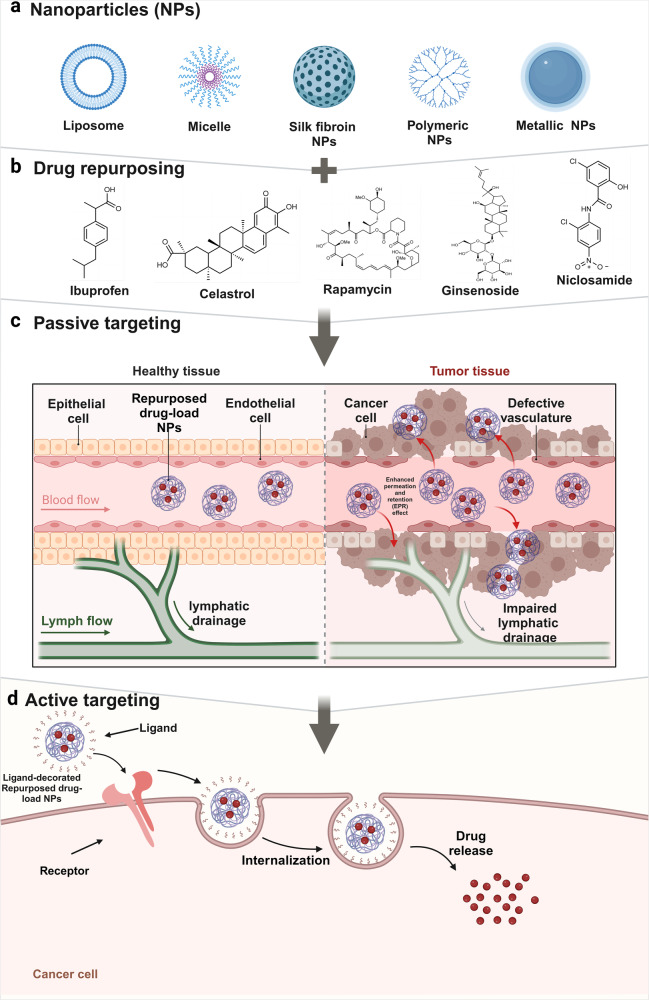
Nanocarriers in repurposed drug delivery in cancer. Multiple nanocarriers including polymeric nanocarriers, mesoporous silica nanoparticles, polymeric micelles, liposomes, and metallic nanoparticles have shown promising effects in the delivery of repurposed therapeutics in cancer. Targeted cancer NP drug delivery systems: **a** Diversity of NPs in thedrug delivery platform. **b** Active-targeting ligand-decorated repurposed drug-loaded NPs for cancers. **c** Passive tumor targeting via the enhanced permeability and retention (EPR) effect. **d** Internalization of active-targeting NPs driven by receptor activation. This figure was created with Biorender.com

The efficacy of repurposed therapeutic agents is chiefly determined by their side effects and resistance profiles.^345^ While the ultimate goal is to pinpoint and precisely target cancer cells, prevailing systemic drug administration techniques struggle with challenges such as uneven distribution and suboptimal pharmacokinetics, which can translate into unwanted side effects. Moreover, the non-specific nature of drug delivery might result in a disproportionate accumulation of repurposed agents in organs such as the kidneys, liver, and spleen. Such uneven distribution often culminates in inadequate drug concentrations at the tumor site, thereby reducing the drug’s potential to effectively inhibit tumor growth.^346^ Thus, it is crucial to devise the right therapeutic strategies to enhance their impact on tumor growth, while minimizing side effects and drug resistance. Here, our emphasis lies primarily on nano-delivery systems for topical drug delivery, such as polylactic-co-glycolic acid (PLGA) nanoparticles, mesoporous silica nanoparticles, polymeric micelles (PMs), liposomes, and metallic nanoparticles (Fig. 5). These nanoparticles have demonstrated their potential for drug delivery and imaging in biomedicine.^347^ Nanoparticles can minimize the systemic toxicity of traditional therapies, thereby significantly enhancing the ability of repurposed drugs to eliminate tumors without harming normal cells.^348^ In this section, we catalog the various nano-carriers tailored for the targeted delivery of repurposed drugs against tumors, alongside ongoing clinical trials that are using nanomaterials to treat cancer.

### Polymeric nanoparticles

PLGA polymers have emerged as a focal point in drug delivery research due to their^349^ outstanding biodegradability and biocompatibility, as well as their easy modifiability for precise drug control and release.^350,351^ This adaptability is particularly impactful when encapsulating drugs and elevating their targeted delivery, especially in oncology. For example, Güor e et al. found a significant reduction in cellular proliferation in A-549 cells treated with ibuprofen-loaded unique EudragitVR RS 100 and/or octadecylamine modified PLGA nanoparticles compared to cells treated with the equivalent dosage of unencapsulated drug. Such outcomes accentuate the transformative potential of PLGA nanoparticles in augmenting cancer therapeutics.^352^

### Silk fibroin nanoparticles

Silk fibroin (SF), derived from silkworms, is a biocompatible and biodegradable protein, making it an ideal candidate for various biomedical applications. Its unique scaffolding and matrix properties have been shown to exhibit exceptional cell compatibility both in vivo and in vitro.^353,354^ Given these attributes, SF is gaining traction as a potential material for drug delivery systems, and could therefore play a transformative role in treating a variety of diseases. One example of the potential application of SF involves celastrol, a bioactive compound extracted from the traditional Chinese herb, clover. Celastrol possesses a distinct chemical structure and a range of bioactivities including its anticancer properties. However, its direct clinical use has been limited by its poor water solubility and marked toxicity.^355,356^ To circumvent these challenges, Ding et al. innovatively encapsulated celastrol into SF nanoparticles (CL-SFNPs) using a modified desolvation method and found that CL-SFNPs outperformed free-form celastrol in terms of antitumor efficacy, inhibition of colony formation, and induction of cancer cell apoptosis.^354^ Thus, these findings highlight the potential of SFNPs to act as a delivery platform for celastrol to advance cancer therapies.

### Polymeric micelles

PMs were one of the first polymeric self-assemblies and possess many advantages over other nano-assemblies^357^ including ease of processing, a streamlined architecture, and superior drug solubilization. In addition, PMs exhibit enhanced biocompatibility, advanced pharmacokinetics, biodistribution properties, and increased adaptability for engineering customizations.^358,359^ As a result, PMs are excellent vehicles for drug delivery, displaying better absorption and reduced side effects through varied administration methods, which range from parenteral to oral, nasal, and ocular routes.^360–363^ Thus, PMs could act as potential carriers for repurposed drugs. A case in point is rapamycin (RAPA), a naturally-derived macrolide drug lauded for its anti-fungal, anti-angiogenesis, and immunosuppressive traits. In order to exploit the full potential of RAPA, Shin et al. pioneered the development of fenbendazole/rapamycin-loaded mPEG-b-PCL micelles (M-FR) via freeze-drying. Notably, the M-FR system was found to increase the cytotoxicity and reduce the proliferation abilities of ovarian cancer cells compared to treatment with the unconjugated fenbendazole/rapamycin combination. Moreover, in vivo pharmacokinetic studies revealed that M-FR offered improved bioavailability and significant antitumor effects.^352^ This collaborative application of a nanocarrier system, therefore, has the potential to re-engineer RAPA, initially developed as a non-tumoricidal drug, into a formidable tumoricidal agent, thus making it a potent strategy in the chemoprevention of cancer.

### Liposomes

Liposomes are lipid bilayer nanovesicles designed for the transport of diverse molecules such as DNA, proteins, antigens, and drugs.^364^ Their inherent ability to transport both hydrophobic and hydrophilic agents,^347^ combined with their ease of synthesis, extensive surface area, and adaptability for targeted modifications, positions them as a pivotal vehicle for drug delivery.^365^ Niclosamide (NIC), recognized as an anthelmintic drug for over 50 years, also serves as an effective inhibitor of STAT3. NIC has been shown to induce apoptosis in breast, lung, and colon cancer.^366,367^ However, the clinical application of NIC has been constrained due to its poor water solubility and bioavailability. Shah and colleagues addressed this issue using a design-of-experiments approach to develop NIC-loaded liposomes, which were found to enhance antitumor efficacy against melanoma cells in vitro.^368^ Similarly, Hatamipour et al. found that NIC-loaded nanoliposomes markedly inhibited the growth of CT26 colon cancer cells, leading to a pronounced delay in tumor progression and increased survival.^369^ These promising outcomes highlight the potential of using a reinvigorated strategy such as NIC-loaded liposomes in cancer therapeutics.

### Metallic nanoparticles

Metallic nanoparticles, notably gold and silver, possess unique properties, such as their small size and large specific surface area, which enhance their surface affinity towards specific antibodies and ligands, and increase their efficiency in targeted drug delivery for various ailments.^370,371^ Their prominence as anticancer therapeutic agents arises from their unparalleled stability, substantial drug-loading capacities, potent targeting capabilities, and precise drug-release mechanisms.^372^ Among repurposed phytochemicals, ginsenoside is of interest to oncologists due to its potential tumor-suppressing properties. However, its therapeutic potential may be limited by the hydrophobic nature of its aglycone backbone, suboptimal bioavailability, and cytotoxicity to benign cells. In an attempt to address these issues, Kim et al pioneered the synthesis of gold nanoparticles carried by *Lactobacillus kimchicus* DCY51T as a potential nano-pharmaceutical against solid tumors. This novel nano-delivery system not only displayed increased cytotoxicity but also increased apoptotic rates within tumors compared to application of ginsenoside alone,^373^ suggesting that metal nanoparticle-imbued repurposed drugs may have the ability to inhibit tumor proliferation and potentially induce regression of established tumors.

## Challenges of drug repurposing

Although systematic drug repurposing has provided new opportunities, to date, few repurposed drugs in cancer or even oncology have successfully transitioned into clinical application. Even though the drug repurposing process is perceived to be significantly quicker and less expensive than traditional drug discovery, rushing into clinical trials could potentially impede the search for more precise treatments. Furthermore, like all drug development, there still exists a risk of failure in the later stages of clinical trials. Other hurdles include legal and regulatory issues, as well as challenges related to pharmacology and dosing.^374,375^ It is our hope that such barriers can be overcome to fully realize the potential of drug repurposing.

### Pharmacological challenges and high effective concentrations may not be clinically achievable

Drug repurposing, while promising, presents several pharmacological challenges. Drugs initially tailored for specific receptors, cells, or organs might not have the same efficacy when rerouted for different therapeutic purposes. Consequently, higher doses or augmented drug interactions might be necessary to achieve therapeutic levels, which could, in turn, introduce novel mechanisms of action, distinct from their intended use.^376^ This shift could lead to unexpected binding to off-target molecules, and the introduction of unforeseen side effects when the drug is administered to humans. Unfortunately, the nuances of dosage and achievable blood serum concentrations are often overlooked, rendering the clinical translation of such drugs problematic. Alterations in dosage can escalate the risk of adverse events. For example, repurposing simvastatin and metformin for cancer could raise questions about their potential to cause hypolipidemia and hypoglycemia, respectively. Likewise, could aspirin repurposed in this manner increase the risk of gastrointestinal bleeding? The feasibility of these repurposed treatments in a clinical setting will depend on the tolerability of side effects during their administration period.

### Patent considerations and regulatory considerations

Intellectual property (IP) rights present another significant issue that needs to be addressed. It is possible to secure IP protection for a newly repurposed medical use of a known drug molecule in most of the major pharmaceutical markets, provided that the new medical use is novel and inventive (i.e., non-obvious). However, many potential repurposing uses are already known in the scientific literature, which limits the potential for securing patent protection for the repurposed context.^58^ In certain cases, even when a drug has demonstrated promising results, its market entry can be hindered due to conflicts associated with IP rights. Furthermore, in cases of repurposing an off-patent drug, the prospect for a return on investment is limited, which makes industries less inclined to fund a trial. In addition to patent issues, there are numerous legal and regulatory barriers to drug repurposing. Regulatory incentives or formal guidance encouraging companies to invest in research and development for further uses of existing drugs are often lacking, or if present, are inadequate.^375^

## Conclusion and future perspectives

Theoretically, repurposed drugs can partially alleviate the shortage of new drugs and resistance to existing chemotherapeutic drugs.^377^ In this review, we have drawn on an expansive exploration of drug repurposing research over the years to collate and appraise potential drug candidates for cancer therapy, the relevant clinical studies of which are presented in Table 3. We have explored the mechanisms by which non-oncological drugs target the hallmarks of cancer and the specialized TME. However, it remains unclear whether these drugs will translate into clinical medications. For patients with advanced disease or chemotherapy resistance who lack alternative treatment options, combination therapy is a promising and valuable treatment option. Combining repurposed therapeutic drugs with approved anticancer drugs can achieve synergy and improve therapeutic effectiveness and safety. In addition, as with other drug strategies for cancer treatment, new drug delivery technologies are necessary for treating cancer cells. In this regard, nanotechnology represents a potential strategy to improve the current prognosis and treatment of tumors.^378^ Currently, large clinical studies are examining the use of established safety nanomedicines such as nab-paclitaxel (Abraxane), liposomal DOX, liposomal verteporfin (Visudine), and gadolinium nanoparticles (AGuIX)^379^ in cancers such as pancreatic cancer,^380^ advanced squamous NSCLC,^381^ breast cancer,^382^ platinum-refractory metastatic urothelial cancer,^383^ and gastrointestinal cancer.^384^ However, many components (materials) of the nanocarriers that have exhibited excellent tumor targeting and therapeutic properties in a xenograft tumor model have not been tested for safety and their long-term toxicity is not known. This has resulted in few clinical trials of nanoparticles loaded with repurposed drugs for cancer treatment. Although these target-based nano-formulation therapies have seen minimal translation from pre-clinical research to clinical usage, the increasing number of studies on the synergistic combination of drugs targeting new mechanisms with traditional therapeutics to eliminate cancer survival pathways suggests that the emerging field of combining repurposed drugs loaded with nanomaterials with first-line anticancer drugs for cancer treatment will attract more attention from researchers in the future.

**Table 3:** Summarized repurposed drug candidates under clinical investigation for the treatment of cancer

Drug Name	Register Trial Code	Phase	Status	Study Type
Disulfiram	NCT01118741	Not Applicable	Completed	Interventional
	NCT02101008	II	Completed	Interventional
	NCT03034135^439^	II	Completed	Interventional
Statins	NCT01525407	II	Completed	Interventional
	NCT01988571^440^	II	Completed	Interventional
	NCT00572468	Not Applicable	Completed	Interventional
	NCT03454529^441^	II	Completed	Interventional
	NCT00656292	IV	Completed	Interventional
	NCT01733953^442^	II	Completed	Interventional
	NCT01527045^443^	II	Completed	Interventional
	NCT00580970	II	Completed	Interventional
	NCT03830164	II	Completed	Interventional
	NCT00335504	II	Completed	Interventional
	NCT00334542	II	Completed	Interventional
	NCT01299038	II	Completed	Interventional
	NCT00723398^444^	Not Applicable	Completed	Interventional
	NCT01220973	II	Completed	Interventional
	NCT00354640	II	Completed	Interventional
	NCT02317016^445^	I	Completed	Interventional
	NCT02093390	I	Completed	Interventional
	NCT00490698	II	Completed	Interventional
	NCT01491958	II	Completed	Interventional
	NCT00462280	II	Completed	Interventional
	NCT00647348^446^	II	Completed	Interventional
	NCT05045638	I	Completed	Interventional
	NCT00853580^447^	II	Completed	Interventional
	NCT00185731	II	Completed	Interventional
	NCT00840177^448^	II	Completed	Interventional
	NCT00529542^449^	II	Completed	Interventional
	NCT02061397	I||II	Completed	Interventional
Epigallocatechin 3-gallate	NCT04177693^450^	I	Completed	Interventional
	NCT00596011^451^	II	Completed	Interventional
	NCT00917735^452^	II	Completed	Interventional
	NCT00253643^453^	Not Applicable	Completed	Interventional
Ascorbic acid	NCT01754987	I|II	Completed	Interventional
	NCT02896907	Early I	Completed	Interventional
	NCT00661544	I|II	Completed	Interventional
	NCT00621023	II	Completed	Interventional
	NCT00469209	I|II	Completed	Interventional
	NCT01898091	II	Completed	Interventional
	NCT02278250^454^	I	Completed	Interventional
	NCT00116142^455^	III	Completed	Interventional
	NCT00328614	I	Completed	Interventional
	NCT01987102	I|II	Completed	Interventional
	NCT02175212	III	Completed	Interventional
Celecoxib	NCT02429427^456^	III	Completed	Interventional
	NCT00081263^457^	II	Completed	Interventional
	NCT00953849	I|II	Completed	Interventional
	NCT00571701	II	Completed	Interventional
	NCT00038103	II	Completed	Interventional
	NCT00581971	I|II	Completed	Interventional
	NCT00503035	II	Completed	Interventional
	NCT00538031^458^	II	Completed	Interventional
	NCT00970502	I|II	Completed	Interventional
	NCT00291694	II	Completed	Interventional
	NCT00112502^459^	II	Completed	Interventional
	NCT00073073^460^	II	Completed	Interventional
	NCT00046839	I|II	Completed	Interventional
	NCT02151448	I|II	Completed	Interventional
	NCT00201773	II	Completed	Interventional
	NCT01220973	II	Completed	Interventional
	NCT00357500	II	Completed	Interventional
	NCT00466505	II	Completed	Interventional
	NCT01158534	II	Completed	Interventional
	NCT00499655	II	Completed	Interventional
	NCT02484664^461^	II	Completed	Interventional
	NCT00101686	II	Completed	Interventional
	NCT00033371^462^	II	Completed	Interventional
	NCT03403634	II	Completed	Interventional
	NCT00504660	II	Completed	Interventional
	NCT00582660	II	Completed	Interventional
	NCT01021215	I|II	Completed	Interventional
	NCT00061893^463^	II	Completed	Interventional
	NCT00698204^464^	II	Completed	Interventional
	NCT03084536	II	Completed	Interventional
	NCT00099047	II	Completed	Interventional
	NCT00314262	I|II	Completed	Interventional
	NCT00463060^465^	I|II	Completed	Interventional
	NCT01678313	II	Completed	Interventional
	NCT03031938	III	Completed	Interventional
	NCT04081389	I	Completed	Interventional
	NCT00846430	II	Completed	Interventional
	NCT00927485	Not Applicable	Completed	Interventional
	NCT02073435	III	Completed	Observational
Metformin	NCT01312467	II	Completed	Interventional
	NCT02176161	II	Completed	Interventional
	NCT01433913^466^	II	Completed	Interventional
	NCT00930579^467^	II	Completed	Interventional
	NCT01340300	II	Completed	Interventional
	NCT01447927^468^	II	Completed	Interventional
	NCT02376166	Not Applicable	Completed	Interventional
	NCT02028221^469^	II	Completed	Interventional
	NCT01620593^466^	II	Completed	Interventional
	NCT01579812^470^	II	Completed	Interventional
	NCT02325401^471^	I	Completed	Interventional
	NCT01666730	II	Completed	Interventional
	NCT03109873	Early I	Completed	Interventional
	NCT01310231^472^	II	Completed	Interventional
	NCT01302379^473^	Not Applicable	Completed	Interventional
	NCT01101438^474^	III	Completed	Interventional
	NCT02431676^475^	II	Completed	Interventional
	NCT03117517	Early I	Completed	Interventional
	NCT02949700^476^	I|II	Completed	Interventional
	NCT02022007^477^	III	Completed	Interventional
	NCT00703508	Not Applicable	Completed	Interventional
	NCT01410604^478^	IV	Completed	Interventional
	NCT02498522	Early I	Completed	Interventional
	NCT01877564	II	Completed	Interventional
	NCT00670800^479^	Not Applicable	Completed	Interventional
	NCT01319994^480^	II|III	Completed	Interventional
	NCT01909141^480^	Early I	Completed	Interventional
	NCT01968317	II	Completed	Interventional
	NCT01427595^481^	Not Applicable	Completed	Interventional
	NCT00151411^482,483^	II	Completed	Interventional
	NCT00283816	III	Completed	Interventional
	NCT02635386^484^	III	Completed	Interventional
	NCT00714233	III	Completed	Interventional
	NCT02060383^485^	IV	Completed	Interventional
Curcumin	NCT00094445	II	Completed	Interventional
	NCT01246973	II|III	Completed	Interventional
	NCT00113841	Not Applicable	Completed	Interventional
	NCT01042938	II	Completed	Interventional
	NCT00927485	Not Applicable	Completed	Interventional
	NCT01740323	II	Completed	Interventional
	NCT00641147^486^	II	Completed	Interventional
	NCT02556632	II	Completed	Interventional
	NCT00365209	II	Completed	Interventional
Pump proton inhibitors	NCT01322633		Completed	Observational
	NCT03531762	I	Completed	Interventional
	NCT02595372	II	Completed	Interventional
	NCT02224053^487^	I	Completed	Interventional
	NCT00204373^488^	IV	Completed	Interventional
	NCT03708211^489^	I	Completed	Interventional
	NCT01844583	I	Completed	Interventional
	NCT03219723		Completed	Observational
	NCT00474903^490^	II	Completed	Interventional
	NCT03028103	I	Completed	Interventional
	NCT00656968	IV	Completed	Interventional
	NCT00210470	II	Completed	Interventional
	NCT01848457^491^	II	Completed	Interventional
*β*-adrenergic antagonists	NCT02420223^492^	Not Applicable	Completed	Interventional
	NCT00624416	II	Completed	Interventional
	NCT02740127	I|II	Completed	Interventional
	NCT01951950	III	Completed	Interventional
	NCT01847001	I	Completed	Interventional
	NCT02177175^493^	II	Completed	Interventional
	NCT01358968	II	Completed	Interventional
	NCT03513757	I	Completed	Interventional
	NCT01009918^494^	IV	Completed	Interventional
	NCT02525718	II	Completed	Interventional
	NCT00471445	II	Completed	Interventional
	NCT01678313	III	Completed	Interventional
	NCT02550795	IV	Completed	Interventional
	NCT02550795	IV	Completed	Interventional
	NCT00516503	Not Applicable	Completed	Interventional
	NCT02342275^495^	III	Completed	Interventional
	NCT02913612^496^	III	Completed	Interventional
	NCT01056341^497^	II	Completed	Interventional
	NCT01908972^498^	II|III	Completed	Interventional
	NCT01908972^498^	IV	Completed	Interventional
	NCT03186638	II	Completed	Interventional
Ibuprofen	NCT02351700^499^	IV	Completed	Interventional
	NCT01238120	II	Completed	Interventional
	NCT02227316	IV	Completed	Interventional
	NCT02001974^500^	I	Completed	Interventional
	NCT02480114	III	Completed	Interventional
	NCT00327340^501^	II	Completed	Interventional
	NCT01985763^502^	I|II	Completed	Interventional
Genistein	NCT01325311	II	Completed	Interventional
	NCT00290758^503^	II	Completed	Interventional
	NCT00118040^504^	II	Completed	Interventional
	NCT00244933	II	Completed	Interventional
	NCT00376948	II	Completed	Interventional
	NCT01036321	II	Completed	Interventional

Data obtained fromclinicaltrial.gov (Accessed on 6th Nov 2023)

Drug repurposing has, to date, attracted considerable attention from researchers and pharmaceutical industries worldwide.^385^ Several promising strategies based on clinical symptoms, genome and transcriptome data, as well as various databases, have been devised to advance the development of repurposed drugs for tumor treatment.^386^ This article provides a summary of the most frequently employed approaches to drug repurposing, including both experimental and computational methodologies. The emergence of cutting-edge technologies has resulted in the production of a significant amount of data that includes genomics, proteomics, drug-disease associations, drug chemical structure profiles, and phenotypes. These substantial data resources are pivotal in comprehending the intricacies of cancer mechanisms and enabling large-scale screening of repurposed drugs. Widely used data resources have been compiled and are presented in Table 3 for further reference.

However, approved drugs are encumbered by legal and safety liabilities, IP rights issues, patent and regulatory barriers, and the relative lack of funding for clinical trials by pharmaceutical companies due to expected low returns on investment. Moreover, phase II and phase III clinical trials of repurposed drugs also require substantial resources in terms of money and time.^387^ Clinical trials are essential for addressing questions about whether different pharmacodynamic and pharmacokinetic properties are required for their activity in specific settings also remain.^388^

In addition, several key points warrant particular attention. Due to the multiple mechanisms of resistance and complex oncogenic signaling pathways of cancers, monotherapy may be relatively ineffective for cancer patients. This may explain why few repurposed drugs can be used in cancer treatment, and why, in the era of precision medicine, drug combination therapies are a more promising strategy. Drug combination therapies typically target multiple mechanisms, including downstream off-target, parallel pathways, or compensatory signaling. They can also provide alternative strategies for apoptosis-resistant cancers by targeting other modes of cell death such as ferroptosis and pyroptosis. Concurrently, with the rapid development of molecular profiling, the use of non-oncological drugs that have the potential to target multiple hallmarks of cancer and specialized TME could be a vital complement to personalized/precision treatment in the near future. Furthermore, combining repurposed drugs with first-line anticancer drugs will offer cancer patients new treatment opportunities.

Furthermore, taking into account the commonalities and differences between various cancers can also enhance research in drug repurposing. The commonality among different cancers lies in their potential sharing of key molecular and cellular mechanisms. For instance, many types of cancers display dysregulation in cell metabolism,^389,390^ and impaired apoptotic pathways.^391^ In terms of drug repurposing, this implies that certain drugs might show efficacy across multiple cancer types, especially those targeting these common mechanisms. For example, simvastatin, a statin class drug for lowering blood lipids, works by competitively inhibiting the enzyme HMG-CoA reductase, and blocking the synthesis of cholesterol, an end product of mevalonate metabolism.^392^ Cancers such as gastric cancer, pancreatic cancer, and colon cancer, which exhibit dysregulated lipid metabolism and rely on the mevalonate pathway, highlight the potential of repurposing statin drugs for treating various cancers.^393–395^ However, the diversity of cancers is equally important. Different cancer types have distinct genetic backgrounds, biomarkers, and microenvironment interactions, leading to varying responses to the same drug. For example, EGFR inhibitors show significant efficacy in NSCLC with specific EGFR mutations but are less effective in cancers without these mutations.^396^ Therefore, when considering drug repurposing, the specific characteristics and needs of each cancer type must be taken into account.

Moreover, in the process of drug repurposing, it is necessary to conduct in-depth studies of the pharmacodynamics and pharmacokinetics of drugs to determine optimal dosages, administration frequencies, and potential drug interactions. For instance, drugs originally used for cardiovascular diseases might require dosage adjustments when repurposed for cancer treatment to maximize their antitumor effects while minimizing toxic side effects.^13,397^ In addition, the design and execution of clinical trials are crucial for drug repurposing strategies. These include not only traditional assessments of drug safety and efficacy but also a deep understanding of the mechanisms of drugs in specific cancer types. By designing clinical trials that include various cancer types and patient groups, we can more comprehensively evaluate the potential of repurposed drugs.

In summary, drug repurposing offers significant opportunities in the field of cancer treatment, and despite the many challenges, these efforts are undoubtedly worthwhile. Drug repurposing provides a window of opportunity for drug discovery and represents a developing trend in cancer treatment. Despite the numerous challenges, these efforts are undoubtedly worthwhile.^398,399^
